# Clinical Decision Analysis of Genetic Evaluation and Testing in 1013 Intensive Care Unit Infants with Congenital Heart Defects Supports Universal Genetic Testing

**DOI:** 10.3390/genes15040505

**Published:** 2024-04-18

**Authors:** Benjamin M. Helm, Stephanie M. Ware

**Affiliations:** 1 Department of Medical & Molecular Genetics, Indiana University School of Medicine, Indianapolis, IN 46202, USA; bmhelm@iu.edu; 2 Department of Epidemiology, Indiana University Fairbanks School of Public Health, Indianapolis, IN 46202, USA; 3 Department of Pediatrics, Indiana University School of Medicine, Indianapolis, IN 46202, USA

**Keywords:** clinical utility, inpatient cardiovascular genetics, decision curve analysis, screening performance

## Abstract

Extracardiac anomalies (ECAs) are strong predictors of genetic disorders in infants with congenital heart disease (CHD), but there are no prior studies assessing performance of ECA status as a screen for genetic diagnoses in CHD patients. This retrospective cohort study assessed this in our comprehensive inpatient CHD genetics service focusing on neonates and infants admitted to the intensive care unit (ICU). The performance and diagnostic utility of using ECA status to screen for genetic disorders was assessed using decision curve analysis, a statistical tool to assess clinical utility, determining the threshold of phenotypic screening by ECA versus a Test-All approach. Over 24% of infants had genetic diagnoses identified (n = 244/1013), and ECA-positive status indicated a 4-fold increased risk of having a genetic disorder. However, ECA status had low–moderate screening performance based on predictive summary index, a compositive measure of positive and negative predictive values. For those with genetic diagnoses, nearly one-third (32%, 78/244) were ECA-negative but had cytogenetic and/or monogenic disorders identified by genetic testing. Thus, if the presence of multiple congenital anomalies is the phenotypic driver to initiate genetic testing, 13.4% (78/580) of infants with isolated CHD with identifiable genetic causes will be missed. Given the prevalence of genetic disorders and limited screening performance of ECA status, this analysis supports genetic testing in all CHD infants in intensive care settings rather than screening based on ECA.

## 1. Introduction

Congenital heart disease (CHD) represents the most prevalent class of birth defects, with at least 20–30% of individuals having identifiable genetic causes at present [1,2]. Genetics evaluations, including assessment by medical geneticists and genetic testing, are recommended, though several studies confirm underutilization of these services for patients with CHD [2,3]. There is a wide spectrum of genetic etiologies, including several hundred monogenic and cytogenetic disorders associated with CHD per a recent query of the *Online Mendelian Inheritance in Man* (OMIM). Genetic diagnosis of CHD requires substantial and broad knowledge of genetic disorders and various genetic testing strategies and results alter medical management [1,4,5]. Despite increasing knowledge of genetic causes of CHD—including chromosomal aneuploidies, chromosome copy-number variation (CNV), and monogenic disorders—genetic testing is underused and screening for genetic disorders is unstandardized [2,6,7,8,9,10,11,12,13,14,15]. Several recent studies have shown the benefits of standardizing genetics evaluations in hospitalized patients with CHD [16,17,18,19]. Improving early diagnosis of genetic disorders can inform diagnosis-specific medical management, individual/family risk assessment, and genetic counseling [4,20,21].

One of the primary challenges in ascertaining genetic disorders results from variable disease expressivity and heterogeneous clinical presentations of CHD. Additionally, genetic causes of CHD are often individually rare, leading to low population-attributable risk on a CNV-, gene-, and/or disorder-specific basis [22]. This challenges genetic testing strategies that are based on narrow phenotype-specific diagnostic differentials, knowledge of individual providers, or screening strategies in the most obviously high-risk patients. CHD may present as apparently isolated CHD without overt syndromic dysmorphic features or extracardiac anomalies (ECAs), in addition to CHD plus multiple congenital anomalies (i.e., CHD plus ECAs). Studies show that >20% of those with CHD plus ECAs have genetic diagnoses, and it is likely that this has influenced biased screening approaches for genetic disorders in medically complex CHD patients over those with isolated CHD [2,15,22]. Similarly, infants with genetic syndromes may initially present as apparently isolated CHD due to variable or age-dependent ECA expression and age-related onset of recognizable dysmorphic features. This would lead to underdiagnosis of genetic disorders in apparently isolated/non-syndromic CHD where clinical genetic testing practices vary, despite studies showing that 6–10% of isolated CHD patients have genetic diagnoses [1,16]. There are stronger recommendations for phenotype-guided genetic testing for patients with CHD plus ECAs, but ECA status and/or syndromic presentations can be difficult to ascertain in young, critically ill patients and without clinician dysmorphology expertise [2,22,23,24]. There is less guidance on when to consider genetic testing in apparently isolated CHD and/or CHD without ECAs. The performance of phenotype-guided screening vs. broader population genetic testing for genetic disorders in CHD remains uninvestigated. ECA-positive status is associated with 2- to 3-fold increased risk of genetic disorders [17,25]; however, it has not been subjected to screening performance assessment following standardized genetic testing for all CHD. We hypothesize that clinical programs that prioritize evaluations using ECA status to assess risk of genetic disorders will miss a substantial number of diagnoses. This would result in delayed diagnosis and missed care opportunities, especially in young patients in the ICU setting.

Our study leverages a unique inpatient Cardiovascular Genetics program that has standardized inpatient clinical genetics evaluations in CHD patients since 2014, with genetic testing for nearly all CHD inpatients [16]. At minimum, this has included chromosome microarray analysis for all patients ± gene panels or exome sequencing; the program has now evolved to complete genome sequencing as the standard, beginning in 2022. This provides an opportunity to assess performance of ECA status as a screen for genetic disorders compared to the standard of genetic testing for all patients with/without ECAs. Here we focus on the analysis of this protocol for infants less than 12 months of age admitted to the ICU. Results will help define genetic testing strategies for this population with CHD and allow assessment of ECA status to screen patients for risk of having a genetic disorder. Our study goals include the following: (1) quantify the association of ECA status with genetic diagnoses identified and describe prevalence of genetic diagnoses in ECA-negative and ECA-positive infants in the ICU; (2) identify anatomic-specific ECA patterns associated with CHD classes—overall and for those with genetic diagnoses identified; and (3) assess the performance of ECA status as a screen for genetic disorders and include decision curve analysis to define the net benefit of its use compared to testing all patients with CHD regardless of ECA status.

## 2. Methods

### 2.1. Overview, Study Design, and Ethics

This retrospective study used a patient cohort from our inpatient Cardiovascular Genetics program at Riley Hospital for Children at Indiana University Health (2014–2023). The cohort consists of mostly neonates and infants admitted with CHD and referred for inpatient genetics evaluation, including physical examinations by board-certified medical geneticists, genetic counseling, and coordination of genetic testing during the inpatient admission. This and similar inpatient CHD genetics programs have been described previously [16,17]. We receive consultations for almost all admitted patients with CHD in the neonatal intensive care unit and cardiovascular intensive care unit (NICU/CICU), and we standardized genetic testing practices across consulting geneticists. Internal quality assessment suggests that >95% of these patients completed genetics evaluations during admission from 2019–2023. This study was deemed exempt after review by the Indiana University Institutional Review Board (IRB protocol #17818).

### 2.2. Subjects and Case Classifications

The cohort sample includes any neonate or infant inpatient with CHD referred to the Cardiovascular Genetics service; no CHD cases were excluded. We used complete-case data for all analyses. Early in the program, we generally did not receive consults for trisomy 21, but from 2018–present, referrals increased for suspected trisomy 21 requiring clinical evaluations and confirmatory genetic testing. Each CHD was classified as one of eight mutually exclusive categories based on the definitions by the National Birth Defects Prevention Study [26]. These CHD classes included anomalous pulmonary venous return (APVR), atrioventricular septal defects (AVSD), complex, conotruncal, heterotaxy/laterality spectrum defects, left ventricular outflow tract obstructions (LVOTO), right ventricular outflow tract obstructions (RVOTO), and septal categories (also known as Level 3 categories). Use of the “complex” class was reserved for CHD representing multiple classes and defying mutually exclusive categorization. Consultations with medical geneticists included physical examination, dysmorphology evaluation, documentation of ECA status, and recommended diagnostic genetic testing. ECA status was defined dichotomously (absent/present) to include any noncardiac major anomalies, including structural anomalies/malformations as well as medically significant functional anomalies (e.g., seizures, dystonia, growth restriction, immunodeficiency, and hypo/hypercalcemia). ECAs are defined as major anomalies with medical/cosmetic significance and differentiated from minor anomalies (dysmorphisms) using previous guidelines [27,28]. We also classified each ECA occurring in patients as representing specific body systems or organs. ECA status occurred at the time of each consultation, generally preceding availability of genetic testing results, by manual review of the inpatient medical records and clinical genetics consultation notes. The classic definition of genetic syndrome as a recognizable pattern of traits or medical diagnoses that tend to occur together was utilized by medical geneticists in their assessment of infants. At the time of consultation, each patient was assessed as having a confirmed genetic syndrome, a possible genetic syndrome, or no syndrome/isolated CHD. Patients with confirmed syndromes at evaluation either met clinical criteria for diagnosis given their recognizable features (e.g., Down syndrome or Noonan syndrome), which was later confirmed by genetic testing, or had genetic testing results already available and an evaluation which confirmed the diagnosis.

### 2.3. Genetic Testing Practices and Defining Genetic Diagnoses

Three phases define our minimum genetic testing strategies in the inpatient CHD genetics program, namely: (1) chromosome microarray (CMA) ± additional molecular genetic testing based on geneticist evaluations (or diagnostic prenatal genetic testing) (2014–2019); (2) CMA plus exome sequencing (ES) or ES-based gene panels (2020–2022); and (3) genome sequencing (GS) ± additional genetic testing at the discretion of the genetics team (2022–present). Genetic testing was performed by Clinical Laboratory Improvement Amendments (CLIA) and College American Pathology (CAP)-certified commercial genetic testing laboratories in the United States. Molecular genetic testing, including phenotype-specific gene panels, exome-based gene panels, and exome sequencing/genome sequencing (singleton, duo, or trio samples) were performed by GeneDx, Inc. (Gaithersburg, MD, USA), Prevention Genetics, Inc. (Marshfield, WI, USA), and Baylor Genetics Clinical Diagnostics Laboratory (Houston, TX, USA) using standard methods (Supplemental Methods in Supplemental Materials). From 2019 to 2022, ES-based methods used copy-number variant (CNV) identification in next-generation sequencing data, confirmed by CMA (Prevention Genetics, Inc.). Genome sequencing also included CNV calling (Baylor Genetics Clinical Diagnostics Laboratory). Genetic testing results were classified as (1) normal, (2) variant(s) of uncertain significance (VUSs), or (3) diagnostic (i.e., pathogenic or likely pathogenic results), following the American College of Medical Genetics and Genomics (ACMG)/Association for Molecular Pathology (AMP) guidelines for variant interpretation [29]. Results were classified as diagnostic (yes vs. no) for pathogenic/likely pathogenic variants that (a) confirmed a genetic/syndromic diagnosis and/or (b) causally explained the CHD phenotype with confidence. Secondary findings were not investigated in this study. When a genetic diagnosis was identified, results were classified as cytogenetic, molecular genetic (monogenic), cytogenetic and molecular diagnoses (>1 coinciding diagnoses), or clinical diagnosis (with uninformative genetic testing).

### 2.4. Additional Data

We recorded basic demographic and clinical information including sex assigned at birth, gestational age (when available), age at consult (in days), age group (neonate or infant), parent-reported race/ethnicity for the patient as recorded in the electronic health record, birth measurements (weight in grams, length in centimeters, and head circumferences in centimeters, when available), maternal diabetes status (verified by documentation of pregestational or gestational diabetes when able), genetic testing ordered/completed, number of genetic tests completed, and outcomes of genetic testing. We also recorded mortality status and age at death for non-surviving cases. Generally, all demographic and clinical variables in this study were recorded in the context of the patient admission, i.e., ranging from one week to three months or more for longer care courses.

### 2.5. Statistical Analyses and Screening Performance

Descriptive statistics are presented for relevant demographic and clinical variables using proportions (%) for categorical data and mean and median for continuous data (with standard deviation and lower/upper quartile range, respectively). For some variables, missing data led to slightly varying denominators, and no imputation methods were used. When testing for the primary aims of this study, only complete case analysis was used, i.e., cases with non-missing ECA status and genetic diagnosis. We then tested for differences in ECA status and genetic diagnosis identified across other variables using chi-squared tests of independence (Χ^2^)/Fisher exact tests for categorical data and the Kruskal–Wallis test for non-normal continuous data. When applicable, we also report odds ratios (OR) with 95% confidence intervals. We investigated organ- and system-specific ECA patterns across CHD classes using tetrachoric correlation. Estimates of strength of correlation coefficient were fair (±0.3–0.5), moderate (±0.6–0.7), and very strong (>±0.8) [30]. We assessed the performance of ECA status as a screen for genetic disorders later diagnosed/confirmed by genetic testing. For these aims, we report screening performance using sensitivity/specificity, accuracy, and Youden index (*J*)/number needed to diagnose (NND) defined as 1/*J*. We also provide positive and negative predictive values reported as percentages (PPV and NPV, respectively) and the predictive summary index (PSI) as a metric of overall prediction (PPV + NPV-1 and equivalently as PPV-false-negative rate) [31]. We included the clinical utility indices (CUIs) and used these values to calculate the summary utility index (SUI); suggested values for poor, adequate, and good performance were based on the literature [32,33]. We estimated the OR for ECA-positive status using logistic regression, and we report the associated Brier score and area-under-the curve (AUC) for the receiver operator characteristic curve. Screening and classification performance metrics are summarized in tabular form. Last, we assessed for differences in genetic diagnosis over the three time periods of our program using Cochran–Armitage one-sided trend tests and stratified by ECA status. A statistical significance threshold of *p* < 0.05 was used. We used SAS 9.4 for all analyses (SAS Institute, Cary, NC, USA).

### 2.6. Decision Curve Analysis

To investigate the clinical utility of using ECA status to predict/screen patients at higher risk of having genetic disorders, we performed decision curve analysis, an approach with established usage for determining the net benefit of implementing a screening/prediction model for determining interventions in healthcare [34,35,36,37]. Such analyses evaluate whether a model helps support clinical decisions and which model leads to the best decisions [36]. Interventions can be considered across a range of patient- or clinician-acceptable risk thresholds, considering the benefits and costs/harms of under- or over-treatment (or under- or over-diagnosis). Decisions about the “intervention” can include ordering a diagnostic medical test or not (e.g., diagnostic genetic testing based on ECA status) [35]. The key metric for decision curve analysis is net benefit (NB), defined as NB = TPR*_R_* P − [*R*/(1 − *R*)] FPR*_R_* (1 − P), where “high risk” is defined as risk above some risk threshold, *R*, considering the true/false positive (TP/FP) rates of a screen/model and prevalence of the disease/outcome of interest (P). Costs/harms and benefits of the interventions are implicit in decision curve analysis, but these do not have to be explicitly modeled [37]. The analysis also helps assess the ability of a risk model to correctly classify and assign risks for patient outcomes considering the prevalence of genetic disorders in our cohort (in this case, risk of genetic diagnoses based on ECA status). Decision curve analysis classically calculates the net benefit of three scenarios when assessing a single screening test or model: Intervention for All, Intervention for None, and Using the Model to Guide Decisions about Intervention. In the context of this study, our three scenarios include: (1) Test All, (2) Test None, and (3) Use ECA Status to Determine Genetic Testing Decisions. Our goal is to determine the clinical utility of ECA status to screen CHD patients for being at high risk of having a genetic disorder, compared to the Test-All strategy agnostic to ECA status. Results are summarized in the form of a decision curve, as defined in guidelines [34,35,36,37]. We used the Decision Curve Analysis SAS macro to complete the analysis for this study (https://www.danieldsjoberg.com/dca-tutorial/dca-tutorial-sas.html [accessed 1 November 2023]); this was created by Daniel J. Sjoberg with additional contributions by Shaun Porwal and Andrew Vickers, and is available on GitHub: https://github.com/ddsjoberg/dca-tutorial [accessed 1 November 2023]).

## 3. Results

### 3.1. Cohort Description

The study cohort comprised n = 1013 neonates and infants, described in more detail in Table 1. Males were more prevalent than females (56.3% and 43.7%, respectively), with a median age at consult of 3 days. Several races/ethnicities were represented, including 12.6% Black/African American, 10.7% Latino/Hispanic, and 69.8% White. The most common CHD classes included LVOTO (25.7%) followed by conotruncal (24.6%), septal (13.4%), and complex (13.0%). Nearly 43% of patients were classified as ECA-positive, including ≥1 ECAs as defined in this study. However, most patients were classified as having apparently isolated/non-syndromic CHD (65.8%). Nearly 24% of patients had possibly syndromic CHD, followed by the minority of patients having genetic diagnoses confirmed at/by physical exam (10.3%). Maternal pregestational or gestational diabetes was reported for 8.4% of patients. Additional stratification of key study variables across ECA status and genetic diagnosis identified is described in Table 2 and Table 3.

### 3.2. Genetic Testing and Genetic Diagnostic Outcomes

The majority of patients completed recommended genetic testing, with only 2.2% of the cohort not having any genetic testing ordered/completed (Table 1). Otherwise, the most common genetic testing strategies included exome-sequencing/ES-based testing with CMA (37.5%), followed by CMA only (28.8%) and genome sequencing (20.7%). Other tests included single-gene testing, gene panels, and other cytogenetic tests, i.e., chromosome karyotype and FISH. Some patients were admitted based on diagnostic genetic testing completed prenatally or at outside facilities. Overall, 24.1% of patients had a genetic diagnosis identified, specified further by 16.6% having cytogenetic disorders and 6.9% with molecular genetic (monogenic) disorders. Genetic diagnostic yields varied across genetic testing types. When limited to CMA, ES/ES-based testing with CMA, and genome sequencing, diagnostic yields were 20.9%, 15.0%, and 20.5%, respectively (*p* = 0.0929). Excluding the AVSD class, prevalence of genetic diagnoses was highest in the septal (33.8%), complex (25.0%), conotruncal (25.3%), and RVOTO classes (21.4%); diagnoses were the least prevalent among LVOTO, APVR, and heterotaxy classes (Table 3). We found genetic diagnoses in all CHD classes. Diagnostic proportions differed minimally when excluding trisomy 21.

### 3.3. ECA Status and Genetic Diagnoses Identified

ECA-positive status was associated with several variables (Table 2). As expected with potentially age-dependent ECA status, more infants had ECA ascertained compared to neonates (*p* = 0.0024); it is also possible that this is influenced by referrals of more complex CHD patients to our center’s neonatal heart center or level 4 NICU. ECA was most prevalent in the septal, AVSD, and heterotaxy CHD classes (*p* < 0.0001). Patients with possibly syndromic and syndromic clinical presentations had higher proportions of ECA-positive status compared to apparently isolated/non-syndromic CHD patients (*p* < 0.0001). Patients with a genetic diagnosis identified were more likely to have ECA-positive status compared to those without a genetic diagnosis (68.0% vs. 32.0%, *p* < 0.0001). While there were more males than females in this cohort, more females had genetic diagnoses identified compared to males (27.5% vs. 21.4%, *p* = 0.0235). There were no differences in the proportion of genetic diagnoses identified across age groups (*p* = 0.5190) and race/ethnicity (*p* = 0.8930), providing evidence that our program has equitable testing and diagnosis. Otherwise, genetic diagnoses were identified less frequently in ECA-negative (13.5%) compared to ECA-positive patients (*p* < 0.0001). Interestingly, it was less common to identify genetic diagnoses in patients with a history of maternal diabetes (14.1% vs. 25.0%, *p* = 0.0247). 

### 3.4. ECA Patterns and Associations with Genetic Diagnoses Identified

**Figure 1:**
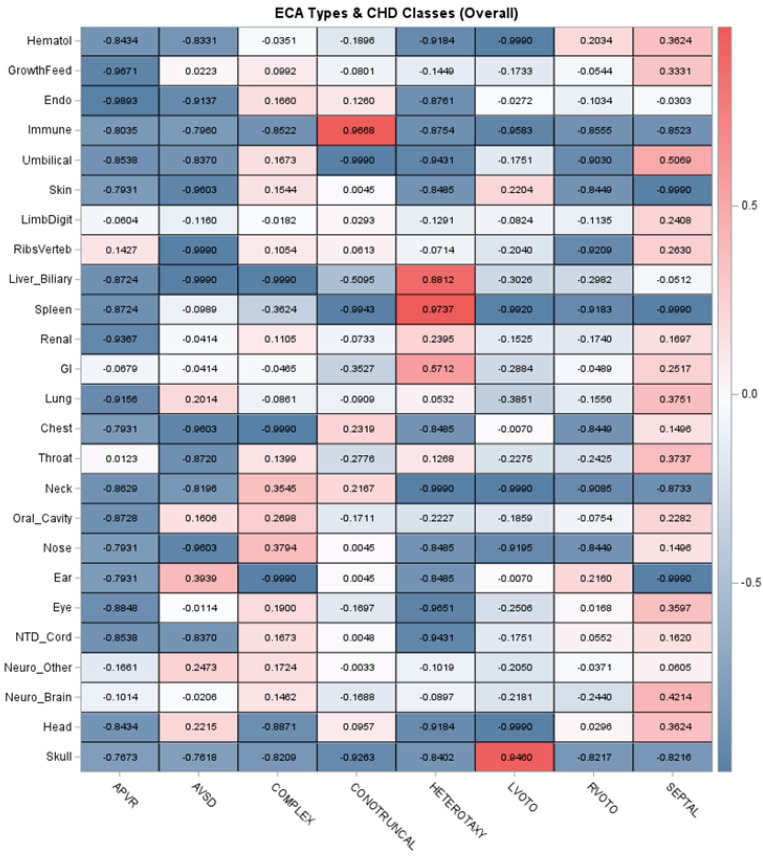

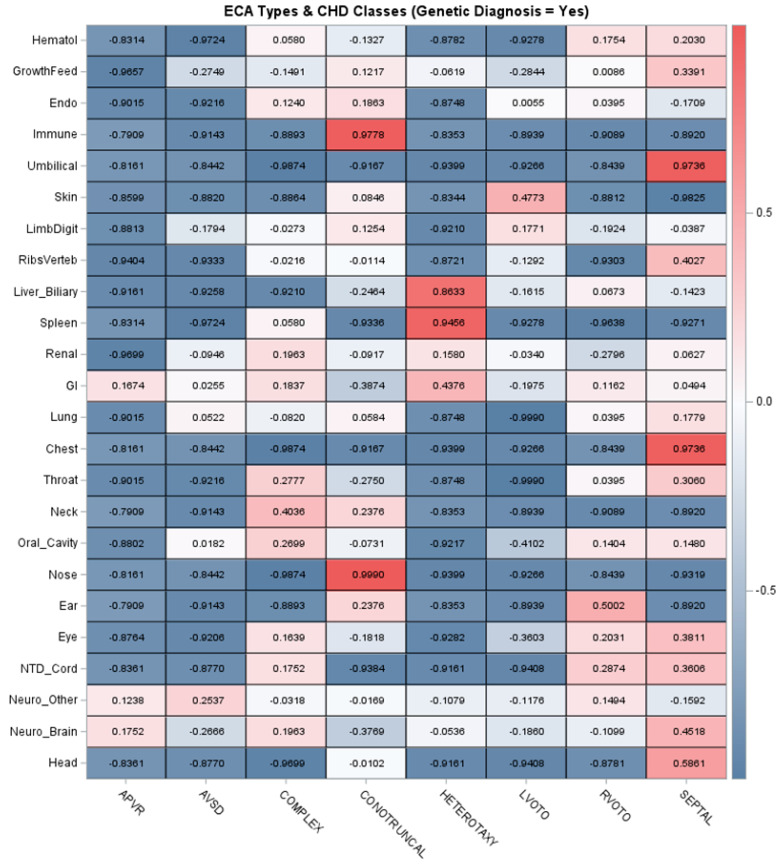
Correlations between organ- or system-specific extracardiac anomalies and classes of congenital heart disease. Note: The top panel depicts the correlations across the entire cohort, and the bottom panel summarizes the correlations in patients with a genetic diagnosis identified. The strength of correlation is indicated by the color intensity. Acronyms: Endo = Endocrine, GI = Gastrointestinal/Abdominal Wall, Hematol = Hematology, NTD = Neural Tube Defect.

Table 4 summarizes associations between different ECA types and identifying genetic diagnoses. Overall, ECAs were individually uncommon, with most organ- or system-specific ECAs occurring in ≤4% of patients. The most prevalent ECA types were gastrointestinal/abdominal wall (9.6%), renal (9.6%), neurological—brain (5.9%), neurological—functional (8.1%), and growth/feeding abnormalities (7.4%). Despite the low prevalence of individual ECA types, there were multiple associations with genetic diagnoses identified. The strongest associations were reflected in statistically significant ORs ranging from ≥3 to ≥5 and included skin, neurological, eye, oral cavity, limb/digit, endocrine, and hematologic (Table 4). Other ECA types had ORs ranging from >1 to 3, including some outliers with very wide confidence intervals. These results show that some ECA types are more strongly associated with genetic disorders than others. We then investigated correlation patterns between ECA types across CHD classes, including cohort-wide correlations and stratification by patients with a genetic diagnosis identified (Figure 1). Figure 1 shows CHD correlations with a range of ECA types for the complex and septal classes. This pattern may be influenced by medical complexity for these two CHD classes, especially patients with medically complex septal CHD requiring intensive care. The heterotaxy class had relatively strong associations with gastrointestinal, renal, spleen, and liver/biliary ECAs, as expected. When restricted to only patients with genetic diagnoses identified, the complex, conotruncal, RVOTO, and septal classes had wider ranges of ECA correlations approximately ≥0.20. Interestingly, there were weak or negative correlations in LVOTO patients with genetic diagnoses, suggesting this class is enriched for genetic disorders which may not have ECAs and/or clinically present as apparently isolated/non-syndromic CHD (Figure 1). 

### 3.5. Screening Performance of ECA Status

Screening metrics and predictive performance of ECA status are summarized in Table 5. The PPV for ECA status was 38.3% and the NPV was 86.6%. These values are influenced by the prevalence of genetic diagnoses in ECA-positive and -negative patients, as well as the number of diagnoses found in apparently isolated/non-syndromic CHD (Table 1, Table 2 and Table 3). The sensitivity and specificity of ECA status were 0.68 and 0.65, respectively, and the overall accuracy was 0.66. As an overall assessment of screening performance, the Youden index (*J*) was 0.33, suggesting moderately accurate classification (*J* ranges from −1 to 1, with 0 not offering any benefit and 1 indicating perfect performance). The related NND was 3.0, suggesting that three patients would need to be examined to correctly identify one truly affected case. The predictive summary index (PSI) indicates a net gain of 24.9% in predictive certainty when using ECA status. Using logistic regression, ECA-positive status was associated with a 4-fold increased risk of genetic diagnoses identified (OR = 4.0 [2.9, 5.4], *p* < 0.0001). Despite this relatively strong association, ECA status has a low–moderate classification performance based on key metrics (e.g., AUC = 0.667, Brier score 0.17). The PSI supports an incremental, but low–moderate, improvement in prediction when using ECA status. However, PSI is dependent on disease prevalence, and values of 50–70% would be considered ideal with our study prevalence of genetic disorders [38]. The summary utility index measure suggests that ECA status has poor to adequate performance (SUI = 0.8258); however, there is higher utility for ECA-negative status based on CUI_(−)_ of 0.5650, which is considered adequate [33].

### 3.6. Decision Curve Analysis

**Figure 2:**
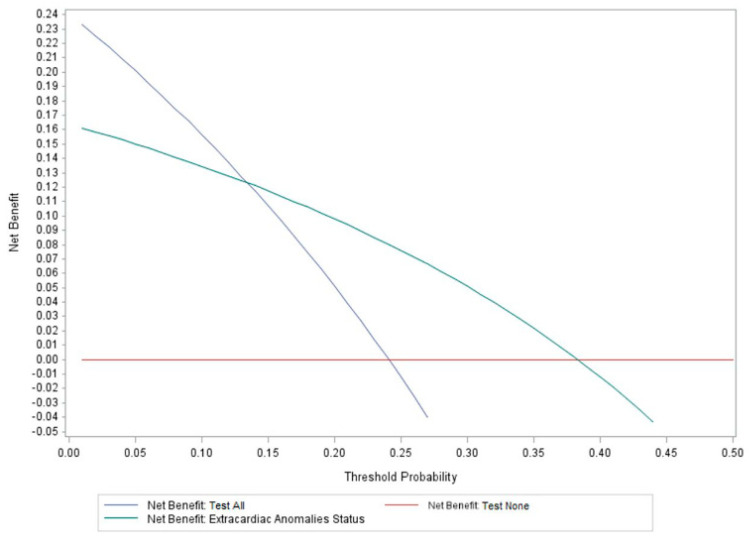
Results of the decision curve analysis summarizing use of extracardiac anomaly status to screen for genetic disorders. vs. the Test-All and Test-None alternatives. The highest net benefit for using ECA status to screen for high risk of genetic disorders in patients occurs at a risk threshold of ≥14%; however, the Test-All net benefit is higher when the risk threshold is <14%.

Results of the decision curve analysis are summarized in Figure 2. Use of ECA status as a predictor of genetic disorders in CHD patients has a higher net benefit across a range of risk thresholds, specifically ≥14–40%. However, its net benefit is lower than the Test-All strategy in the risk range of <14% (Figure 2). The decision curves cross each other at a risk threshold of 14%, and this corresponds approximately to the prevalence of genetic diagnoses identified in ECA-negative patients in this cohort (13.5%, Table 3). This indicates that using ECA status to screen for risk of genetic disorders would result in higher net benefit, but only when clinicians would consider risks of >14% to be considered “high risk” enough to warrant genetic testing. For example, in Figure 2, if a clinician suggests there needs to be at least a 20–25% chance of a genetic disorder occurring in a patient with CHD before they would consider genetic testing, then use of ECA status would be preferred. However, if a clinician suggested that there needs to be at least a 5–10% chance of a genetic disorder occurring, then the Test-All strategy would have the highest net benefit since it would maximize the true-positive rate compared to use of ECA status. Based on the decision curve, risks of ≤14% should use the Test-All approach. Given the prevalence of genetic diagnoses identified in ECA-negative patients (13.5%) and in apparently isolated/non-syndromic cases (8.1%) (see Table 3), these results suggest that genetic testing in all CHD cases in the ICU would have a higher net benefit compared to strictly using ECA status as a screen to triage patients at high risk of genetic disorders. Otherwise, a number of genetic diagnoses would be missed due to their prevalence in isolated/non-syndromic CHD.

### 3.7. Types of Genetic Diagnoses and Informing Future Genetic Testing Strategies

We assessed for possible patterns in types of genetic diagnoses according to ECA status. A list of these diagnoses is available in Supplemental Results S1. We included low-count types of diagnoses like those that had >1 concurrent cytogenetic/molecular genetic diagnosis (n = 5) and clinical diagnoses (n = 2). Of the 245 patients with a genetic diagnosis identified, 78 (31.8%) patients were ECA-negative; more specifically, 57/78 (73.1%) had cytogenetic diagnoses and 20/78 (25.6%) had molecular genetic diagnoses. One case was ECA-negative and had two concurrent genetic diagnoses (1/78, 1.3%). There is no association between ECA status and cytogenetic vs. molecular genetic disorders (Supplemental Results S2, *p* = 0.67) This suggests that strict use of ECA-positive status for ordering genetic testing would result in missing nearly one-third of identifiable genetic conditions (31.8%). Numerous cytogenetic and monogenic disorders were identified patients with or without ECA, and ECA status alone would not sufficiently inform genetic testing strategies, e.g., CMA only testing vs. stepwise CMA plus additional testing. 

### 3.8. Assessing Differences in Genetic Diagnosis over Program Time Periods

**Figure 3:**
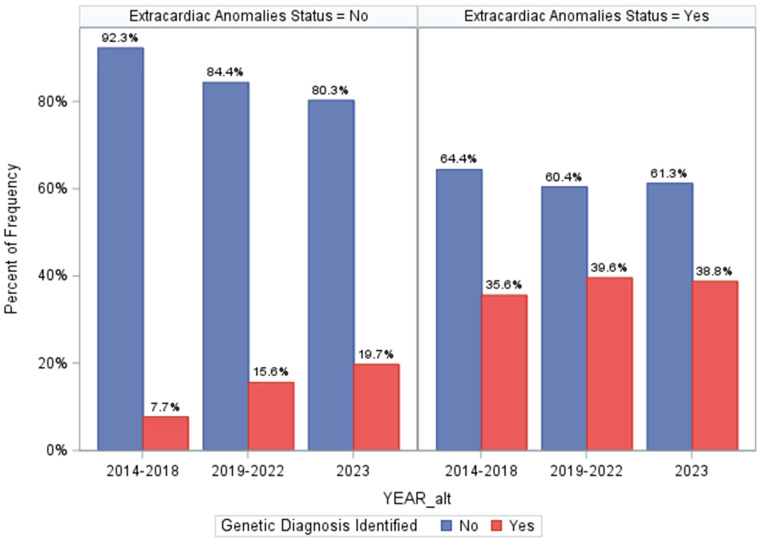

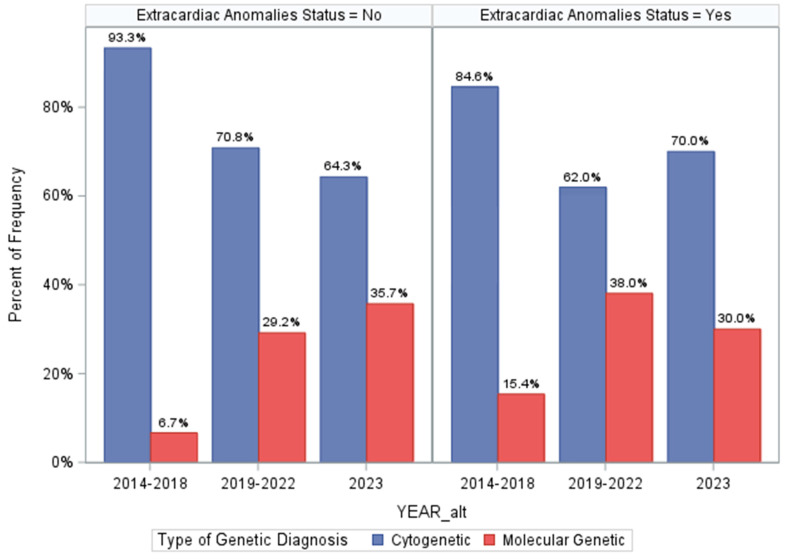
Differences in genetic diagnosis identified across the three time periods of our program and stratified by extracardiac anomaly status. The top panel shows the prevalence of genetic diagnoses overall in each team period, and the lower panel is restricted to comparing prevalence of cytogenetic and monogenic disorders across each time period (using the Cochran–Armitage trend test).

Last, we assessed how differences in genetic testing strategies over the three time periods of our program may have influenced prevalence estimates of genetic disorders (Figure 3 and Supplemental Results S3). This could have also influenced the screening metrics for ECA status. While genetic testing technologies may have differed over time, medical geneticist practices did not—all patients had access to CMA ± additional sequencing-based testing based on their assessments. However, the minimum standards evolved over time from CMA-first priority to CMA plus ES and then GS. Importantly, we found that among ECA-positive patients, there were no differences in diagnostic yields of genetic testing over time (*p* = 0.2994, Figure 3), suggesting that our ECA-specific screening metrics are not influenced substantially by varying disease ascertainment over time. Otherwise, among ECA-negative patients, diagnostic proportions increased over time from 7.7% to 15.6% and eventually to 19.7% (*p* = 0.0015, Figure 3). The same trend was seen when comparing prevalence of cytogenetic and monogenic disorders in the ECA-negative patients over time (Figure 3), with an increasing number of molecular genetic diagnoses identified in the ECA-negative patients. Finally, we found that when controlling for ECA status, there did not appear to be a strong association between identifying genetic diagnoses based on the time period, though it was marginally significant (*p* = 0.0494, Supplemental Results S3). This analysis supports that the screening metrics for ECA status as presented here are not substantially altered by changing technologies, specifically because incremental gains in diagnostic yields occurred only in ECA-negative patients. This lends additional support that ECA status and “medical complexity” are limited for accurately assigning risk of genetic disorders in young CHD patients. 

## 4. Discussion

Current guidelines recommend genetic testing in CHD patients, with those with ECA being considered higher risk [2,39]. As genetic testing technologies have improved and become more widely accessible, guidelines have continued to emphasize their importance for those CHD patients with ECA without directly addressing patients who are ECA negative [39]. It has been demonstrated that practice variation is substantial and that providers caring for CHD patients are more likely to employ genetic testing in those with ECA [3]. However, the screening performance of ECA status has not been investigated. Our inpatient Cardiovascular Genetics program standardized comprehensive genetic testing for all CHD patients, allowing us to measure the performance of ECA status. Our finding that 6–10% of isolated CHD patients have genetic diagnoses replicates our previous findings using a larger cohort and conforms with results from another institution [16,17]. Our study is novel in that more contemporary and comprehensive genetic testing strategies were used that were largely agnostic to ECA status, e.g., combined CMA plus exome-based testing and genome sequencing for all CHD classes [1,2,4,12,16,40]. We found that over 24% of patients in the ICU setting have genetic diagnoses identified, and ECA-positive status (42.7% of patients) was associated with a 4-fold increased risk of genetic disorders occurring. ECA-positive status remains one of the strongest predictors of CHD patients having a genetic disorder. ECA prevalence in our study was higher compared to that in previous studies (~25–30%), and this may be influenced by the enrichment of ECA in patients requiring intensive care, as well as our broader definition, which includes functional abnormalities [41]. However, ECA prevalence has varied widely based on study design and cohort differences, and by review of post-mortem CHD cases. For example, ECA prevalence in CHD ranges within 13–66% in autopsy series and within 9–55% in clinical cohorts [41,42,43,44,45]. A recent study of ECA estimated a prevalence of 54.5%, and these investigators similarly found a high prevalence among patients with septal defects and those with abnormal genetic testing results [45]. Interestingly, we found that a history of maternal gestational or pre-gestational diabetes did not vary in those with or without ECA status. However, genetic diagnoses were less commonly found in patients whose mothers had a history of gestational or pre-gestational diabetes (*p* = 0.0247). While ascertainment bias is possible, and retrospective records are often limited in documenting maternal comorbidities in patient records, 8.4% of the cohort had a history of maternal diabetes. This is in line with population estimates, suggesting a lower risk of under-ascertainment [46,47,48]. Given the association between maternal diabetes and CHD incidence, future investigation of the intersection between maternal diabetes, CHD, and genetic risk factors is warranted.

ECA status has previously been investigated in association with genetic disorders and types of CHD [10,17,25,44,45]. We found additional organ- and system-specific ECA associations with CHD types and genetic diagnoses identified. Relatively strong associations were found for central nervous system, eye, oral cavity, and endocrine anomalies, and this may aid in clinical screening in CHD patients. Similar patterns have been reported by others [45]. Notably, endocrine anomalies included hypo/hypercalcemia, and these can be seen in disorders like 22q11.2 deletion syndrome/DiGeorge syndrome and Williams syndrome [49,50]. Our work also adds to prior descriptions of ECA patterns that may be associated with specific CHD classes [45]. Septal and complex CHD were correlated with a wider range of ECA types, and central nervous system anomalies were also most prevalent across CHD types and in those with genetic disorders. Together, these findings support ECA-CHD patterns described nearly 50 years ago by Greenwood and colleagues [41]. Their work showed that 8.5% of patients had specific recognizable syndromes by clinical exam, similar to our 9.8% [41]. These investigators suggested that the type of ECA could help predict the CHD type (prior to the advent of echocardiography in their case), and similarly our results could help direct screening for ECA types based on CHD class. Last, our estimates are likely conservative given the neonatal patients and potential for under-ascertaining ECA in patients discharged early and/or due to age-dependent penetrance.

Despite the strong association with ECA-positive status in this and previous studies, we determined that ECA status has limited screening performance at a population level. For example, we found that ECA status had a PPV of 38.3% and NPV of 86.6%. Overreliance on ECA-positive status would result in missing 32% of patients found to have genetic diagnoses by genetic testing, and both cytogenetic and molecular genetic disorders were found without ECA present. We also found that changes to the genetic testing algorithm over the time periods of the clinical program likely did not substantially alter assessment of ECA screening performance. More specifically, we saw incremental gains in diagnostic yield over time, but this was only seen for the ECA-negative patients. This suggests that improved identification of genetic disorders cannot be attributed solely to changes in our genetic testing standards, but instead, to increased referrals of isolated CHD for genetics evaluations, as well as wider genetic screening for ECA-negative cases. This adds to our conclusion that ECA-positive status is not an ideal screen for genetic disorders in young CHD patients. There are two primary conclusions from this: (1) ECA status is insufficient for screening for possible cytogenetic vs. molecular genetic diagnoses; in fact, several CNV syndromes with early medical/developmental management implications were identified in ECA-negative patients (Supplemental Results S1); and (2) ECA status is insufficient for determining the best genetic testing strategy, i.e., we found both molecular and cytogenetic disorders across both ECA statuses. This suggests that ideal genetic testing strategies should employ methods that can ascertain the full spectrum of types of genetic diagnoses. Comprehensive genetic testing strategies would be ideal in patients with apparently isolated CHD as well as CHD plus ECAs.

An interesting finding is the prevalence of genetic disorders in ECA-negative LVOTO patients, showing that genetic disorders in this class specifically may present in an isolated fashion, warranting genetic testing. Prior epidemiologic analyses have shown that relatively “strong” risk factors/biomarkers on a risk ratio scale may not translate into good population screens, especially when considering PPV and NPV, limited discrimination (i.e., low–moderate AUC), and prior population prevalence [51]. Decisions to use a risk model for screening must consider the implications of false-negative, i.e., misclassified low-risk, results, e.g., ECA-negative status in this study. ECA can still be useful, however, but the PSI of 24.9% and SUI value show its limitation in improving predictive certainty considering the population prevalence of genetic disorders. Notably, PSI depends on prevalence, and our study prevalence of genetic disorders ranged from 13.5% to 38.3% for ECA-negative and ECA-positive patients, respectively. At these ranges, the PSI of an ideal screen should be 70–90%, and ECA status falls below this [38]. This does not indicate an absence of utility for ECA status, but it shows its limited use for prioritizing patients for genetic testing.

The decision curve analysis indicates higher clinical utility and net benefit of testing all patients with CHD rather than using ECA status for screening, especially when the acceptable risk range is ≤14%. Decision curves are not intended to determine the threshold at which decision making should be based; instead, they help illustrate limitations of models/strategies assuming some accepted risk tolerance threshold. Depending on the outcome of interest and the consequences of clinical actions, clinicians can define a threshold for decision making. For CHD, we can use prevalence to inform decision making accounting for enrichment of genetic disorders in this population, and our study suggests a prevalence of at least 24%. In addition, defining a threshold for decision making in CHD should consider a balance between identifying truly affected cases with a genetic disorder, or conversely, missing diagnoses (false-negative). We found that 13.5% of ECA-negative patients and 8.1% of those described as apparently isolated/non-syndromic had genetic diagnoses identified. Therefore, the minimum prevalence of genetic disorders was 8.1–13.5%, and this is similar to previous studies of apparently isolated CHD [1,14,16]. In this range, the Test-All strategy had a higher net benefit and would be favored according to the decision curve analysis. However, this is assuming that clinicians agree that an acceptable risk threshold of approximately 8–14% is sufficient to order genetic testing for any CHD patient and regardless of the CHD class or ECA status.

Updates to future guidelines for CHD genetic testing should consider results from this study. Additional classification metrics like the Youden index, AUC, and Brier score show that ECA status has low–moderate performance as a screen for genetic disorders. The PSI of 24.9% indicates modest incremental improvement in predictive certainty, supporting results from the decision curve analysis [52]. If the primary goal of inpatient genetics care for CHD patients is to make early diagnoses or to reduce missed diagnostic opportunities, then genetic testing in all CHD patients in the ICU setting would have the highest clinical utility, considering the prevalence of genetic disorders and number of missed diagnoses when relying on ECA-positive status. However, a point of caution is necessary: diagnosing genetic disorders is not necessarily synonymous with improved clinical outcomes in CHD patients; however, these may be defined. Inpatient programs must consider the potential benefits, costs, and limitations of inpatient CHD genetic testing and define specific priorities of genetics evaluations. Some recent studies highlight utility of early genetic diagnoses in the course of CHD inpatient care, especially surrounding perioperative medical management [6,20,21]. 

While the performance of ECA status is limited and decision curve analysis supports the clinical utility of a Test-All approach, our results do not indicate the best-performing, most cost-effective, or ideal genetic testing strategy. This will require future studies of similarly standardized genetic testing for inpatient CHD. Despite this, our results indicate that ECA status does not help prioritize types of genetic testing to consider; chromosomal abnormalities and monogenic disorders were found in all patients, across all CHD classes, and regardless of ECA status. Given the heterogeneous genetic causes of CHD, efficient and comprehensive genetic testing strategies would be ideal. Our program has evolved from CMA ± additional or stepwise gene panels, to CMA plus ES/ES-based panels, and eventually to genome sequencing as the standard. Several studies support the use of CMA and exome sequencing for CHD [10,11,12,14,16,53,54,55,56,57,58]. However, additional research is needed to determine performance and cost-effectiveness of genome sequencing for CHD, and some groups have begun exploring this [59,60,61]. GS has the ability to ascertain a fuller spectrum of CHD genetic etiologies singularly and efficiently—aneuploidies, copy-number variants, monogenic disorders, multiple co-occurring genetic disorders, and non-coding variation previously elusive to gene panels and exome sequencing. As GS costs decrease and availability increases, it will likely become the de facto diagnostic genetic test for CHD and other birth defects in the ICU setting. Given our study’s results, it may be reasonable to consider genome sequencing for all CHD in the intensive care setting.

### Limitations

This study assessed a primarily neonate/infant patient cohort admitted for intensive care, and the results may not be generalizable to the wider CHD population, e.g., those with less complex CHD and/or those with apparently isolated CHD. ECA status was also determined at the time of genetics consultation, and with a median age at consult of 3 days, it is possible for some patients to develop relevant ECAs later in the care course or beyond the data collection period. ECA status may have been challenging to determine without additional imaging or testing due to intubation, surgery, etc., or because of early mortality. Therefore, ECA may be potentially underestimated, although our prevalence is higher than that in previous studies, suggesting this may minimal. This study did not account for ECA-negative patients with ≥1 minor dysmorphism(s), and additional studies are needed to investigate dysmorphology evaluations in ECA-negative patients. The proportion of genetic diagnoses may also be an underestimate in this study given the natural evolution of the program from 2014 to 2023. For example, it may be possible that patients who only had normal initial testing (e.g., CMA) could have diagnoses missed if clinical teams were unable to complete additional stepwise genetic testing, e.g., due to patient death, discharge, or for other reasons. The likelihood of this should have been reduced as our program standardized the CMA plus ES-based test as a standard starting in 2019. It may be possible that some inconclusive genetic testing results (VUS) could be reclassified as causative in the future, resulting in more conservative estimates of genetic disorder prevalence. Similarly, some negative/normal ES/GS results may reflect limited knowledge of novel genes/variants potentially associated with CHD; this highlights the importance of the ability to reanalyze previously negative/inconclusive ES/GS results at follow-up. Subjectivity in variant prioritization and interpretation can occur across genetic testing laboratories, although the risk of this should be minimized with adherence to AMP/ACMG laboratory guidelines. Last, decision curve analysis is a tool to assess population interventions or policies, and it is not intended to determine the benefit of applying model predictions at an individual patient level [37].

## 5. Conclusions

Over 24% of CHD infants in the ICU have genetic disorders identified following implementation of standardized genetic testing—primarily CMA ± exome sequencing/exome-based panels or genome sequencing. Previous research more strongly recommends genetic testing in patients with CHD plus ECAs, and ECA-positive status has been associated with 2- to 4-fold increased odds of identifying a genetic diagnosis. Despite this, we found that ECA status has low–moderate performance as a screen for genetic disorders in the intensive care setting, and 13.5% of ECA-negative patients had genetic diagnoses identified. Decision curve analysis supports higher clinical utility for testing all patients with CHD instead of relying on ECA status, specifically at a risk threshold of ≤14%. This is higher than the prevalence of genetic disorders occurring in apparently isolated/non-syndromic CHD and supports a test-all policy. Of patients with a monogenic or cytogenetic disorder identified, nearly 32% were ECA-negative and would have been missed had ECA-positive status been strictly used to complete genetic testing (i.e., in the most obvious high-risk patients). These findings should help inform the development of inpatient CHD genetics programs and support population genetic screening for CHD in critical care settings. 

## Figures and Tables

**Table 1:** Patient Cohort Description.

Variable	Cohort Descriptive Statistics
**Sex**	
Female	443/1013 (43.7%)
Male	570/1013 (56.3%)
**Age at Consultation (Days)**	Mean = 18.1 (SD = 47.0) Median = 3.0 (IQR: [1.0, 10.0])
**Age Group**	
Neonate (0–28 days)	880/1013 (86.9%)
Infant (29 days–1 year)	133/1013 (13.1%)
**Race/Ethnicity Group** **(Parent-Reported)**	
Asian/Pacific Island	25/1013 (2.5%)
Black/African American	126/1013 (12.4%)
Hispanic/Latino	110/1013 (10.9%)
Other	3/1013 (0.3%)
White	707/1013 (69.8%)
Unknown/Declined	42/1013 (4.2%)
**CHD Class**	
APVR	28/1013 (2.8%)
AVSD	37/1013 (3.7%)
Complex	132/1013 (13.0%)
Conotruncal	249/1013 (24.6%)
Heterotaxy/Laterality Spectrum	73/1013 (7.2%)
LVOTO	260/1013 (25.7%)
RVOTO	98/1013 (9.7%)
Septal	136/1013 (13.4%)
**ECA Status**	
No	580/1013 (57.3%)
Yes	433/1013 (42.7%)
**ECA Number**	Mean = 1.0 (SD = 1.6) Median = 0 ([IQR: [0, 1.0]) Mode = 0
**Maternal Diabetes Status (Gestational & Pregestational)**	
No/Unknown	928/1013 (91.6%)
Yes	85/1013 (8.4%)
**Clinical Description at Evaluation**	
Apparently Isolated/Non-Syndromic	667/1013 (65.8%)
Possibly Syndromic CHD	242/1013 (23.9%)
Confirmed Syndrome at Evaluation	104/1013 (10.3%)
**Consultation Time Period**	
2014–2018	313/1013 (50.9%)
2019–2022	549/1013 (54.2%)
2023	151/1013 (14.9%)
**Genetic Testing Ordering Strategy**	
None	22/1013 (2.2%)
Prenatal Genetic Testing Only	32/1013 (3.2%)
Outside Hospital Genetic Testing	20/1013 (2.0%)
Targeted Cytogenetic Testing Only (FISH, karyotype)	45/1013 (4.4%)
Chromosomal Microarray (postnatal)	292/1013 (28.8%)
Targeted Molecular Genetic Testing Only (phenotype-specific/single-gene)	12/1013 (1.2%)
Chromosome Microarray + Exome-Based Gene Panel/Exome Sequencing	380/1013 (37.5%)
Genome Sequencing	210/1013 (20.7%)
**Number of Genetic Tests Completed**	Mean = 1.5 Median = 1.0 (IQR: [1.0, 2.0]) Mode = 1.0 Range = 4.0
**Genetic Diagnosis Identified/Confirmed**	
No	769/1013 (75.9%)
Yes	244/1013 (24.1%)
**Genetic Testing Result Types**	
Normal/Negative	451/1013 (44.5%)
Inconclusive with ≥1 Variant(s) of Uncertain Significance	319/1013 (31.5%)
Diagnostic	243/1013 (24.0%)
**Genetic Diagnosis Type(s)**	
None/Unclear	768/1013 (75.8%)
Cytogenetic	168/1013 (16.6%)
Molecular Genetic	70/1013 (6.9%)
Cytogenetic & Molecular (>1 Diagnosis)	5/1013 (0.5%)
Clinical Diagnosis, with Uninformative Genetic Testing	2/1013 (0.2%) ‡

‡ Both of these cases were primary ciliary dyskinesia diagnosed based on abnormal ciliary biopsies; one case had completely negative/normal genetic testing, and another case had a single pathogenic variant in an autosomal recessive gene with assumption by the clinical team that a gene-specific genetic diagnosis was made but the other allele was undetectable by the genetic testing at that time. Acronyms: APVR = anomalous pulmonary venous return; AVSD = atrioventricular septal defect; CHD = congenital heart defect; ECA = Extracardiac anomalies; FISH = fluorescence in situ hybridization; IQR = Interquartile Range, LVOTO = left ventricular outflow track obstructive defect; RVOTO = right ventricular outflow tract obstructive defect; SD = standard deviation.

**Table 2:** Descriptive Statistics of Extracardiac Anomalies Status Across Relevant Variables.

	Extracardiac Anomalies Status		
Variable	No	Yes	X^2^/Exact or Kruskal-Wallis Test *p*-Value
**Sex**			
Female	247/443 (55.8%)	195/443 (44.2%)	*p* = 0.3951
Male	333/570 (58.4%)	237/570 (41.6%)	
**Age at Consultation (Days)**	Mean = 13.4 (SD = 36.0) Median = 3.0 (IQR: [1.0, 8.0])	Mean = 24.2 (SD = 58.0) Median = 2.0 (IQR: [1.0, 14.0])	*p* = 0.5462
**Age Group**			
Neonate (0–28 days)	520/880 (59.1%)	360/880 (40.9%)	*p* = 0.0024
Infant (29 days-1 year)	60/133 (45.1%)	73/133 (54.9%)	
**Race/Ethnicity Group** **(Self-Reported)**			
Asian/Pacific Island	13/25 (52.0%)	12/25 (48.0%)	*p* = 0.2229
Black/African American	61/126 (48.4%)	65/126 (51.6%)	
Hispanic/Latino	62/110 (56.4%)	48/110 (43.6%)	
Other	1/3 (33.3%)	2/3 (66.7%)	
White	421/707 (59.6%)	286/707 (40.5%)	
Unknown/Declined	22/42 (52.4%)	20/42 (47.6%)	
**CHD Class**			
APVR	23/28 (82.1%)	5/28 (17.9%)	*p* < 0.0001
AVSD	17/37 (45.9%)	20/37 (54.1%)	
Complex	71/132 (53.8%)	61/132 (46.2%)	
Conotruncal	168/249 (67.5%)	81/249 (32.5%)	
Heterotaxy/Laterality Spectrum	8/73 (11.0%)	65/73 (89.0%)	
LVOTO	187/260 (71.9%)	73/260 (28.1%)	
RVOTO	71/98 (72.5%)	27/98 (27.6%)	
Septal	35/136 (25.7%)	101/136 (74.3%)	
**Maternal Diabetes Status** **(Gestational & Pregestational)**			
No/Unknown	533/925 (57.6%)	392/925 (42.4%)	*p* = 0.2850
Yes	44/85 (51.8%)	41/85 (48.2%)	
**Clinical Description at Evaluation**			
Apparently Isolated/Non-Syndromic	526/667 (78.9%)	141/667 (21.1%)	*p* < 0.0001
Possibly Syndromic CHD	27/242 (11.2%)	215/242 (88.8%)	
Confirmed Syndrome at Evaluation	27/104 (26.0%)	77/104 (74.0%)	
**Consultation Time Period**			
2014–2018	195/313 (62.3%)	118/313 (37.7%)	*p* = 0.0078
2019–2022	314/549 (57.2%)	235/549 (42.8%)	
2023	71/151 (47.0%)	80/151 (53.0%)	
**Genetic Testing Ordering Strategy**			
None	19/22 (86.4%)	3/22 (13.6%)	*p* < 0.0001
Prenatal Genetic Testing Only	6/32 (18.8%)	26/32 (81.3%)	
Outside Hospital Genetic Testing	2/20 (10.0%)	18/20 (90.0%)	
Targeted Cytogenetic Testing Only (FISH, karyotype)	17/45 (37.8%)	28/45 (62.2%)	
Chromosomal Microarray (postnatal)	203/292 (69.5%)	89/292 (30.5%)	
Targeted Molecular Genetic Testing Only (phenotype-specific/single-gene)	4/12 (33.3%)	8/12 (66.7%)	
Chromosome Microarray + Exome-Based Gene Panel/Exome Sequencing	217/380 (57.1%)	163/380 (42.9%)	
Genome Sequencing	112/210 (53.3%)	98/210 (46.7%)	
**Number of Genetic Tests Completed**	Mean = 1.4 (SD = 0.6) Median = 1.0 (IQR: [1.0, 2.0]) Mode = 1.0 Range = 3.0	Mean = 1.6 (SD = 0.7) Median = 2.0 (IQR: [1.0, 2.0]) Mode = 1.0 Range = 4.0	*p* = 0.0001
**Genetic Diagnosis Identified/Confirmed**			
No	502/769 (65.3%)	267/769 (34.7%)	*p* < 0.0001
Yes	78/244 (32.0%)	166/244 (68.0%)	
**Genetic Testing Result Types**			
Normal/Negative	296/451 (65.6%)	155/451 (34.4%)	*p* < 0.0001
Inconclusive with ≥1 Variant(s) of Uncertain Significance	206/319 (64.6%)	113/319 (35.4%)	
Diagnostic	78/243 (32.1%)	165/243 (67.9%)	
**Genetic Diagnosis Type(s)**			
None/Unclear	502/768 (65.4%)	266/768 (34.6%)	Exact *p* < 0.0001
Cytogenetic	57/168 (33.9%)	111/168 (66.1%)	
Molecular Genetic	20/70 (28.6%)	50/70 (71.4%)	
Cytogenetic & Molecular (>1 Diagnosis)	1/5 (20.0%)	4/5 (80.0%)	
Clinical Diagnosis, with Uninformative Genetic Testing	0/2 (0.0%)	2/2 (100.0%)	

**Table 3:** Descriptive Statistics of Genetic Diagnosis Identified Across Relevant Variables.

	Genetic Diagnosis Identified		
Variable	No	Yes	X^2^/Exact or Kruskal-Wallis Test *p*-Value
**Sex**			
Female	321/443 (72.5%	122/442 (27.5%)	*p* = 0.0235
Male	448/570 (78.6%)	122/570 (21.4%)	
**Age at Consultation (Days)**	Mean = 17.0 (SD = 43.5) Median = 3.0 (IQR: [1.0, 10.0])	Mean = 21.6 (SD = 56.6) Median = 2.0 (IQR: [1.0, 10.0])	*p* = 0.0586
**Age Group**			
Neonate (0–28 days)	671/880 (76.3%)	209/880 (23.8%)	*p* = 0.5190
Infant (29 days-1 year)	98/133 (73.7%)	35/133 (26.3%)	
**Race/Ethnicity Group** **(Self-Reported)**			
Asian/Pacific Island	19/25 (76.0%)	6/25 (24.0%)	*p* = 0.8930
Black/African American	97/126 (77.0%)	29/126 (23.0%)	
Hispanic/Latino	84/110 (76.4%)	26/110 (23.6%)	
Other	2/3 (66.7%)	1/3 (33.3%)	
White	532/707 (75.3%)	175/707 (24.8%)	
Unknown/Declined	35/42 (83.3%)	7/42 (16.7%)	
**CHD Class**			
APVR	24/28 (85.7%)	4/28 (14.3%)	*p* < 0.0001 *
AVSD	17/37 (45.9%)	20/37 (54.1%)	
Complex	99/132 (75.0%)	33/132 (25.0%)	
Conotruncal	186/249 (74.7%)	63/249 (25.3%)	
Heterotaxy/Laterality Spectrum	64/73 (87.7%)	9/73 (12.3%)	
LVOTO	212/260 (81.5%)	48/260 (18.5%)	
RVOTO	77/98 (78.6%)	21/98 (21.4%)	
Septal	90/136 (66.2%)	46/136 (33.8%)	
**ECA Status**			
No	502/580 (86.6%)	78/580 (13.5%)	*p* < 0.0001
Yes	267/433 (61.7%)	166/433 (38.3%)	
**Maternal Diabetes Status** **(Gestational & Pregestational)**			
No/Unknown	694/925 (75.0%)	231/925 (25.0%)	*p* = 0.0247
Yes	73/85 (85.9%)	12/85 (14.1%)	
**Clinical Description at Evaluation**			
Apparently Isolated/Non-Syndromic	613/667 (91.9%)	54/667 (8.1%)	*p* < 0.0001
Possibly Syndromic CHD	155/242 (64.1%)	87/242 (36.0%)	
Confirmed Syndrome at Evaluation	1/104 (0.96%) †	103/104 (99.04%)	
**Consultation Time Period**			
2014–2018	256/313 (81.8%)	57/313 (18.2%)	*p* = 0.0084
2019–2022	407/549 (74.1%)	142/549 (25.9%)	
2023	106/151 (70.2%)	45/151 (29.8%)	
**Genetic Testing Ordering Strategy**			
None	21/22 (95.5%)	1/22 (4.5%)	*p* < 0.0001 **
Prenatal Genetic Testing Only	11/32 (34.4%)	21/32 (65.6%)	
Outside Hospital Genetic Testing	5/20 (25.0%)	15/20 (75.0%)	
Targeted Cytogenetic Testing Only (FISH, karyotype)	7/45 (15.6%)	38/45 (84.4%)	
Chromosomal Microarray (postnatal)	231/292 (79.1%)	61/292 (20.9%)	
Targeted Molecular Genetic Testing Only (phenotype-specific/single-gene)	4/12 (33.3%)	8/12 (66.7%)	
Chromosome Microarray + Exome-Based Gene Panel/Exome Sequencing	323/380 (85.0%)	57/380 (15.0%)	
Genome Sequencing	167/210 (79.5%)	43/210 (20.5%)	
**Number of Genetic Tests Completed**	Mean = 1.5 (SD = 0.7) Median = 1.0 (IQR: [1.0, 2.0]) Mode = 1.0 Range = 4.0	Mean = 1.5 (SD = 0.7) Median = 1.0 (IQR: [1.0, 2.0]) Mode = 1.0 Range = 3.0	*p* = 0.2799

* When excluding trisomy 21, AVSD remained the class with largest proportion with a genetic diagnosis identified (n = 11/28, 39.3%); there remained a difference across all CHD classes (*p* = 0.0030). † This was a primary ciliary dyskinesia (PCD) clinical diagnosis case confirmed by nasal ciliary biopsy who otherwise had negative genetic testing. A specific genetic diagnosis was not made using genetic testing. The other case of PCD (see footnote in Table 1) was found to have a heterozygous variant and the clinical genetics team considered this diagnostic (while the other allele was assumed to be deeply intronic). ** When limiting genetic testing to CMA-only, CMA plus ES-based panel/ ES, and GS, there was no major difference in diagnostic yields. Proportions of those with diagnostic results include CMA-only (20.9%) CMA plus ES-based panel/ES (15.0%), and GS (20.5%), with *p* = 0.0929.

**Table 4:** Associations Between Organ- or System-Specific Extracardiac Anomalies (ECA) and Genetic Diagnosis Identified.

ECA Type	ECA Cohort Prevalence (%)	Genetic Diagnosis Identified (%)	Exact *p*-Value *	Odds Ratio (95% CI) **
Skull	1/1013 (0.1%)	0/0 (0.0%)	*p* = 1.000	
Head	9/1013 (0.9%)	4/9 (44.4%)	*p* = 0.2309	
Neurological— Brain	60/1013 (5.9%)	33/60 (55.0%)	***p* < 0.0001**	**4.3 [2.5, 7.3]**
Neurological— Other/Functional	82/1013 (8.1%)	40/82 (48.8%)	***p* < 0.0001**	**3.4 [2.1, 5.4]**
Neural Tube Defect/Spinal Cord	8/1013 (0.8%)	4/8 (50.0%)	*p* = 0.1007	
Eye	29/1013 (2.9%)	19/29 (66.5%)	***p* < 0.0001**	**6.4 [2.9, 14.0]**
Ear	4/1013 (0.4%)	2/4 (50.0%)	*p* = 0.2462	
Nose	4/1013 (0.4%)	2/4 (40.0%)	*p* = 1.000	
Oral Cavity	42/1013 (4.0%)	23/42 (54.8%)	***p* < 0.0001**	**4.1 [2.2, 7.7]**
Neck	2/1013 (0.2%)	2/2 (100.0%)	*p* = 0.0578	
Throat—including Esophagus/Trachea	34/1013 (3.4%)	10/34 (29.4%)	*p* = 0.4228	
Chest	4/1013 (0.4%)	1/4 (25.0%)	*p* = 1.000	
Lung	22/1013 (2.2%)	10/22 (43.5%)	***p* = 0.0239**	**2.7 [1.2, 6.3]**
Gastrointestinal & Abdominal Wall	97/1013 (9.6%)	34/97 (35.1%)	***p* = 0.0120**	**1.8 [1.2, 2.8]**
Renal	97/1013 (9.6%)	33/97 (34.0%)	***p* = 0.0239**	**1.7 [1.1, 2.7]**
Liver & Biliary	45/1013 (4.4%)	9/45 (20.0%)	*p* = 0.5956	
Spleen	45/1013 (4.4%)	6/45 (13.3%)	*p* = 0.1032	
Ribs & Vertebral	38/1013 (3.8%)	8/38 (21.0%)	*p* = 0.8467	
Limb/Digit	49/1013 (4.8%)	24/49 (49.0%)	***p* = 0.0001**	**3.2 [1.8, 5.8]**
Skin	4/1013 (0.4%)	3/4 (75.0%)	***p* = 0.0455**	**9.5 [1.0, 92.0]**
Umbilical	8/1013 (0.8%)	1/8 (12.5%)	*p* = 0.6878	
Immunologic	3/1013 (0.3%)	2/3 (66.7%)	*p* = 0.1458	
Endocrine	17/1013 (1.7%)	10/17 (58.8%)	***p* = 0.0021**	**4.7 [1.8, 12.4]**
Growth & Feeding	75/1013 (7.4%)	34/75 (45.3%)	***p* < 0.0001**	**2.9 [1.8, 4.6]**
Hematologic	9/1013 (0.9%)	6/9 (66.7%)	***p* = 0.0080**	**6.4 [1.6, 25.9]**
Other	18/1013 (1.8%)	6/18 (33.3%)	*p* = 0.4029	

* Comparing proportions of those with a genetic diagnosis identified when the specific ECA is present vs absent. Statistically significant results indicate differences in genetic diagnosis proportion compared to the cohort average (24.1%; Table 1). ** The OR was only estimated for statistically significant associations. Statistically significant results and OR are in bold.

**Table 5:** Screening Performance Metrics of Extracardiac Anomalies (ECA) Status for Genetic Diagnoses Identified in Congenital Heart Disease.

Variable	Genetic Diagnosis (n)		Predictive Values (%) for ECA Status	Clinical Utility Index (CUI) for ECA Status (Positive and Negative)
ECA Status	Yes	No		
Yes	166	267	ECA_(+)_ = 0.3834 (38.3%)	CUI_(+)_ = 0.2608 *
No	78	502	ECA_(−)_ = 0.8655 (86.6%)	CUI_(−)_ = 0.5650 *
			PSI = 0.2489 (24.9%) **	SUI = 0.8258 †
**Screening Metrics of ECA Status**				
Sensitivity	0.6803			
Specificity	0.6528			
Accuracy	0.6594			
Youden Index	0.3331			
Number Needed to Diagnose (NND)	3.00			
**Evaluation of ECA Status as** **Predictor of Genetic Diagnoses**				
	Wald X^2^ (*p*-value)	Odds Ratio [95% CI]	AUC [95% CI]	Brier Score
ECA Status (positive vs. negative)	78.2 (*p* < 0.0001)	4.0 [2.9, 5.4]	0.667 [0.633, 0.700]	0.168

* CUI: Clinical Utility Index for positive and negative ECA status (CUI_(+)_ and CUI_(−)_, respectively); CUI_(+)_ is calculated by [sensitivity*PPV] and CUI_(−)_ is calculated by [specificity*NPV]. † SUI: Summary Utility Index = ΣCUI_(+, −)_ ** Predictive Summary Index (PSI), an indicator for net gain in certainty of prediction: PPV + NPV-1. A PSI of 24.9% indicates that ECA status adds incrementally to predictive capability, though it is low to moderate. The number needed to predict (NNP) = 1/PSI = 4.0.

## Data Availability

Requests for data access can be reviewed with the corresponding author, and data can be made available within the confines of ethical review and intended use.

## Supplementary Materials

The following supporting information can be downloaded at: https://www.mdpi.com/article/10.3390/genes15040505/s1, Supplemental Methods, Supplemental Results S1: List of Genetic Diagnoses by Extracardiac Anomaly Status and Genetic Test Type; Supplemental Results S2: Proportion of Cytogenetic and Molecular Genetic Disorders across ECA status; Supplemental Results S3: Assessing differences in genetic diagnosis prevalence over the three time periods of our program.
