# Supplementary material for: Clinical Decision Analysis of Genetic Evaluation and Testing in 1013 Intensive Care Unit Infants with Congenital Heart Defects Supports Universal Genetic Testing
Source: Genes (Basel). 2024 Apr 18;15(4):505. doi: 10.3390/genes15040505 (PMC11050575; doi:10.3390/genes15040505)
Supplement: Supplementary file 1 [file genes-15-00505-s001.zip › Supplemental-Methods.pdf]

Supplemental Methods

Supplemental Methods S1: Diagnostic Genetic Testing Methodologies

Indiana University Cytogenetics Laboratory: Chromosome Microarray
CMA was performed on genomic DNA extracted from peripheral blood using the Applied Biosystems CytoScan HD array platform (ThermoFisher Scientific, Carlsbad, CA, USA) consisting of 1,953,246 unique non-polymorphic copy-number probes and 743,304 single nucleotide polymorphism probes spanning the whole genome. The CNVs were analyzed and reported using the NCBI human genome build 37.1 (GRCh37/hg19) by board-certified cytogeneticists.

Indiana University Cytogenetics Laboratory: Karyotype & FISH
Based on standard methods for rapid FISH for aneuploidy of chromosomes 21, 18, 13, and X/Y. Karyotype used high-resolution banding methods (GTG600).

GeneDx Targeted Exome (XomeSlice)/Next-generations sequencing panels
Methods: Using genomic DNA from the submitted specimen(s), the exonic regions and flanking splice junctions of the genome were captured using a proprietary system developed by GeneDx and sequenced by massively parallel (NextGen) sequencing on an Illumina sequencing system with 100 bp or greater paired-end reads. Reads were aligned to human genome build GRCh37/UCSC hg19, and analyzed for sequence variants in the selected genes or regions of interest using a custom-developed analysis tool (Xome Analyzer). Capillary sequencing or another appropriate methods was used to confirm all potentially pathogenic variants identified in this individual and relative samples, if submitted. Sequence and copy number alterations were reported according to the Human Genome Variation Society (HGVS) and International System for Human Cytogenetic Nomenclature (ISCN) guidelines, respectively.

GeneDx Whole Exome Sequencing
Methods: Genomic DNA was extracted directly from the submitted specimen or, if applicable, from cultured fibroblasts. The DNA was enriched for the complete coding regions and splice site junctions for most genes of the human genome using a proprietary capture system developed by GeneDx for next generation sequencing with CNV calling (NGS-CNV). The enriched targets were simultaneously sequenced with paired-end reads on an Illumina platform. Bi-directional sequence reads were assembled and aligned to reference sequences based on NCBI RefSeq transcripts and human genome build GRCh37/UCSC hg19. Using a custom-developed analysis tool (XomeAnalyzer), data were filtered and analyzed to identify sequence variants and most deletions and duplications involving three or more coding exons (PMID: 25356966). Smaller deletions or duplications may not be reliably identified. Reported variants were confirmed, if necessary, by an appropriate orthogonal method in the proband and, if submitted, in selected relatives. Sequence variants are reported according to the Human Genome Variation Society (HGVS) guidelines. Copy number variants are reported based on the probe coordinates, the

coordinates of the exons involved, or precise breakpoints when known. Reportable variants include pathogenic variants and likely pathogenic variants. Variants of uncertain significance, likely benign and benign variants, if present, are not routinely reported. Available evidence for variant classification may change over time and variant(s) may be reclassified according to the ACMG/AMP Standards and Guidelines (PMID: 25741868), which may lead to issuing a revised report.

Prevention Genetics CAP/CDDP

RAPID CONGENITAL ANOMALIES PANEL WITH CNV DETECTION

Background: PGxome is PreventionGenetics' whole exome sequencing (WES) assay. The PGxome assesses almost all genes from the human genome including coding regions and adjacent introns. This test is intended for health care providers who are looking for a genetic diagnosis. This is important as more than 50% of patients with genetic diseases are not given a specific diagnosis even after repeat clinical examinations and tests (Shashi et al. 2014). The standard clinical practice often involves examinations for specific phenotypes, imaging, biochemical testing for inborn errors of metabolism, genomic tests such as karyotyping or microarrays, and single gene or panel tests (Iglesias et al. 2014). However, patients remain without a genetic diagnosis, and patients and health care providers are caught in a long term diagnosis search, known as a diagnostic odyssey. This can lead to failures in identifying potential treatments, and unknown recurrence and prognosis risks (Yang et al. 2013).
Reported diagnostic rates from commercial and academic laboratories have found that WES assays have a ~20-40% positive diagnostic rate, with higher rates being reported from trio analysis (i.e. proband and parents) compared to singleton analysis (Atwal et al. 2014; Iglesias et al. 2014; Farwell et al. 2015). Notably, ~5-7% of individuals who have WES have had dual diagnoses (i.e. two non-overlapping clinical presentations) (Yang et al. 2014; Farwell et al. 2015; Posey et al. 2016). One study reported that 30% of genetics diagnoses have only been recently resolved due to new literature reports, highlighting the fast pace of gene-disease discovery and the need of genetic testing laboratories to be current of the medical literature (Yang et al. 2014). The use of a whole exome sequencing test may aid in altering clinical management, predict recurrence and prognosis risks, and reduce costs of additional testing, and may offer advantages over traditional molecular tests in certain patients (Valencia et al. 2015, Stark et al. 2017).
Test Methods: For the PGxome we use Next Generation Sequencing (NGS) technologies to cover the coding regions of targeted genes plus ~10 bases of non-coding DNA flanking each exon. As required, genomic DNA is extracted from patient specimens. Patient DNA corresponding to these regions is captured using hybridization probes. Captured DNA is sequenced on the NovaSeq 6000 using 2x150 bp paired-end reads (Illumina, San Diego, CA, USA). The following quality control metrics are generally achieved: >97% of target bases are covered at >20x and mean coverage of target bases >100x. Data analysis and interpretation is performed by the internally developed Infinity pipeline. Variant calls are made by the GATK Haplotype caller and annotated using in house software and Jannovar. Common benign, likely benign, and low quality variants are filtered from analysis. Copy number variants (CNVs) are also detected from NGS data. We utilize a CNV calling algorithm that compares mean read depth and distribution for each target in the test sample against multiple matched controls.

Neighboring target read depth and distribution and zygosity of any variants within each target region are used to reinforce CNV calls. All reported CNVs are confirmed using another technology such as aCGH, MLPA, or PCR.
Reporting: Reports will consist of three different sections: Variants in genes known to be associated with phenotype; Variants in genes possibly associated with phenotype; Variants in genes not associated with phenotype but result in a pediatric disorder (if requested)
*www.omim.org
All differences from the reference sequences (sequence variants) are assigned to one of five interpretation categories (Pathogenic, Likely Pathogenic, Variant of Uncertain Significance, Likely Benign and Benign) per ACMG Guidelines (Richards et al. 2015). Pathogenic, Likely Pathogenic and Variants of Uncertain Significance will be reported in the first and second sections. For the third section, only definitive pathogenic and likely pathogenic variants will be reported. Rare and undocumented synonymous variants are nearly always classified as likely benign if there is no indication that they alter protein sequence or disrupt splicing. Likely benign and benign variants are not included in the reports.
Human Genome Variation Society (HGVS) recommendations are used to describe sequence variants (http://www.hgvs.org).
Limitations and Other Test Notes: Interpretation of the test results is limited by the information that is currently available. Better interpretation should be possible in the future as more data and knowledge about human genetics and this specific disorder are accumulated.
Sequencing: When sequencing does not reveal any heterozygous differences from the reference sequence, we cannot be certain that we were able to detect both patient alleles. Occasionally, a patient may carry an allele which does not capture or amplify, due for example to a large deletion or insertion. In these cases, the report may contain no information about the second allele.
For technical reasons, the PGxome test is not 100% sensitive. Some exons cannot be efficiently captured, and some genes cannot be accurately sequenced because of the presence of multiple copies in the genome. Therefore, a small fraction of sequence variants relevant to the patient's health will not be detected. We sequence coding exons for most given transcripts, plus ~10 bp of flanking non-coding DNA for each exon. Unless specifically indicated, test reports contain no information about other portions of the gene, such as regulatory domains, deep intronic regions, uncharacterized alternative exons, chromosomal rearrangements, repeat expansions, and mitochondrial genome variants.
In most cases, we are unable to determine the phase of sequence variants. In particular, when we find two likely causative variants for recessive disorders, we cannot be certain that the variants are on different alleles. Our ability to detect minor sequence variants due to somatic mosaicism is limited. Sequence variants that are present in less than 50% of the patient's nucleated cells may not be detected. Runs of mononucleotide repeats (eg (A)n or (T)n) with n >8 in the reference sequence are generally not analyzed because of strand slippage during amplification. Unless otherwise indicated, DNA sequence data is obtained from a specific cell-type (usually leukocytes if taken from whole blood). Test reports contain no information about the DNA sequence in other cell-types.
We cannot be certain that the reference sequences are correct. Genome build hg19, GRCh37 (Feb2009) is used as our reference in nearly all cases.

Copy Number Variant Analysis: The PGxome test detects most deletions and duplications including intragenic CNVs and large cytogenetic events; however aberrations in a small percentage of regions may not be accurately detected due to sequence paralogy (e.g., pseudogenes, segmental duplications), sequence properties, deletion/duplication size (e.g., 1-3 exons vs. 4 or more exons), and inadequate coverage. In general, sensitivity for single, double, or triple exon CNVs is ~70% and for CNVs of four exon size or larger is >95%, but may vary from gene-to-gene based on exon size, depth of coverage, and characteristics of the region. Balanced translocations or inversions are only rarely detected. Certain types of sex chromosome aneuploidy may not be detected. In nearly all cases, our ability to determine the exact copy number change within a targeted region is limited. Our ability to detect CNVs due to somatic mosaicism is limited. The sensitivity of this test is dependent on DNA quality.

Baylor MGL Genome Sequencing

Genomic DNA was fragmented and indexed to prepare individuals libraries. The libraries were purified and pooled for sequence analysis on the Illumina NovaSeq platform for paired end reads. Internal quality control measures are performed to ensure sample integrity. Data analysis and interpretation are performed by the Baylor Genetics analytics pipeline. Data are aligned to the human reference genome build GRCh38 using the Illumina Dragen BioIT Platform. Variant calling is performed using the Illumina Dragen haplotype-based variant calling system and Illumina Dragen genome wide depth based CNV (copy number variants) caller with custom modifications from Baylor Genetics. Short tendem repeat calling is performed using the Illumina Mana Structural Variant Caller. The automated genetic interpretation platform from Emedgene is implemented for variant annotation and interpretation with proprietary algorithms and open-source data sets such as gnomAD, EVS (Exome Variant Server), ClinVar, and professional resources such as HGMD (Human Gene Mutation Database) Pro. The variants were interpreted according to ACMG (American College of Medical Genetics) guidelines and patient phenotypes. Variants are confirmed by Sanger sequencing, specific PCR, Multiplex Ligation-dependent Probe Amplification (MLPA), or another methodology, if sequencing or copy number variants detected by NGS do not meet internal quality standards or are within highly homologous regions. Synonymous variants, noncoding variants not affecting splice sites, and common benign variants are excluded from interpretation unless previously reported in the literature as possibly pathogenic. This test may not provide detection of certain variants or portions of certain genes due to insufficient sequencing depth, local sequence characteristics, high/low genomic complexity, or the presence of closely related pseudogenes. Small deletions or insertions larger than 25bp, low-level mosaic variants, structural variants such as inversions, and/or balanced translocations may not be detected with this technology. Variant types being analyzed include sequencing variants and CNV in nuclear genes and mitochondrial DNA (mtDNA), ans short tandem repeats (STR). Uniparental disomy (UPD) analysis is done only in a trio setting. Regions of homozygosity (ROH) are reported with greater than 5 Mb. Sensitivity of detection is decreased for mtDNA sequencing variants and CNVs at heteroplasmy levels lower than 5% and 10%, respectively. Pathogenic events of short tandem repeats can be detected within the genomic regions of following genes: AFF2, AR, ATN1, ATXN1, ATXN2, ATXN3, ATXN7, ATXN10, ATNX80S, C9orf72, CACNA1A, CNBP, CSTB, DIP2B, DMPK, FMR1, FXN, GLS, HTT,

JPH3, NOP56, NOTCH2NLC, PABPN1, PHOX2B, PPP2R2B, RFC1, TBP, and TCF4. Sensitivity of detection is reduced for borderline or incomplete penetrance alleles. The proband report contains results of genes related to the patient's clinical phenotype. Variants relevant to the phenotype are reported as positive (pathogenic/likely pathogenic) or variants of uncertain significance (VUS). Benign and likely benign variants are not reported. This interpretation is based on the clinical information provided by the referring institution and our current understanding of the gene and variants at the time of reporting. Results in this report should be carefully correlated to the individual's clinical findings by the referring physician. If elected, secondary findings including pathogenic and likely pathogenic variants in genes included in the ACMG secondary findings recommendations, or other findings determined to be severe and medically significant (incidental findings), will be reported. This uses the ACMG recommendations. Variants unrelated to the patient's phenotype but potentially clinically significant in genes with no known disease associations will be reported if elected.
