# Supplementary material for: Clinical Decision Analysis of Genetic Evaluation and Testing in 1013 Intensive Care Unit Infants with Congenital Heart Defects Supports Universal Genetic Testing
Source: Genes (Basel). 2024 Apr 18;15(4):505. doi: 10.3390/genes15040505 (PMC11050575; doi:10.3390/genes15040505)
Supplement: Supplementary file 1 [file genes-15-00505-s001.zip › Supplemental-Results_S1-Diagnoses-cyto-molec-eca.pdf]

List of Genetic Diagnoses by Extracardiac Anomaly Status and Genetic Test Type

1. Cytogenetic Diagnoses in Infants with Congenital Heart Defects and No Extracardiac Anomalies

Diagnoses	Frequency	Percent	Cumulative Frequency	Cumulative Percent
15q11.2 deletion (proximal BP1-BP2)	2	3.51	2	3.51
15q11.2 deletion (proximal BP1-BP2) & 1p12 duplication involving portion of NOTCH2	1	1.75	3	5.26
15q11.2 deletion (proximal, BP1-BP2)	1	1.75	4	7.02
16p11.2 deletion syndrome	1	1.75	5	8.77
16p11.2 duplication syndrome	2	3.51	7	12.28
17p13.3 microduplication syndrome	1	1.75	8	14.04
18p11.32-p11.23 duplication & 18q21.32-q23 deletion	1	1.75	9	15.79
1q21 deletion syndrome	1	1.75	10	17.54
1q21.1 duplication syndrome	1	1.75	11	19.30
22q11.2 deletion syndrome	14	24.56	25	43.86
22q11.2 duplication syndrome	1	1.75	26	45.61
3q29 duplication syndrome	1	1.75	27	47.37
6p25.3p25.1 duplication and 9p24.2 duplication and 9q34.3 duplication due to parental balanced translocation	1	1.75	28	49.12
8p23.1 duplication syndrome	2	3.51	30	52.63
Distal 22q11.2 duplication syndrome	1	1.75	31	54.39
Distal 4q- syndrome/Distal monosomy 4q	1	1.75	32	56.14
Hereditary neuropathy with liability to pressure palsies (HNLPP)	1	1.75	33	57.89
Jacobsen syndrome	1	1.75	34	59.65
NODAL-related disorder/heterotaxy visceral 5	1	1.75	35	61.40
Ring chromosome 18 syndrome	1	1.75	36	63.16
Trisomy 18	2	3.51	38	66.67
Trisomy 21	10	17.54	48	84.21
Trisomy 21 (14;21 Robertsonian translocation)	1	1.75	49	85.96
Turner syndrome	4	7.02	53	92.98
Turner syndrome (mosaic with structural X chromosome anomalies)	1	1.75	54	94.74
Turner syndrome (mosaic)	1	1.75	55	96.49

**Williams syndrome**	1	1.75	56	98.25
**Xq28 deletion (BRCC3) moyamoya disease-short stature-facial dysmorphism-hypergonadotropic hypogonadism**	1	1.75	57	100.00

List of Genetic Diagnoses by Extracardiac Anomaly Status and Genetic Test Type

2. Cytogenetic Diagnoses in Infants with Congenital Heart Defects with Extracardiac Anomalies

Diagnosis	Frequency	Percent	Cumulative Frequency	Cumulative Percent
11q25 deletion and 19q13.31q13.43 duplication due to parental balanced translocation	1	0.91	1	0.91
15q11.2 deletion (proximal, BP1-BP2)	1	0.91	2	1.82
15q11.2q26.3 Duplication (partial trisomy 15q)	1	0.91	3	2.73
15q25 microdeletion syndrome/Diamond-Blackfan anemia (RPS17)	1	0.91	4	3.64
15q25.3q26.3 Deletion (13.9 Mb) due to parental balanced translocation	1	0.91	5	4.55
16p11.2 deletion syndrome	3	2.73	8	7.27
16p13.3 deletion syndrome/alpha-thalassemia-intellectualdisability syndrome linked to chromosome 16	1	0.91	9	8.18
1p36 deletion syndrome	2	1.82	11	10.00
1p36 deletion syndrome & 11p15 duplication syndrome (Beckwith-Wiedemann syndrome) due to unbalanced translocation (from parental balanced translocation carrier)	1	0.91	12	10.91
1p36 deletion syndrome (with large 13q33.1-q34 duplication) resulting from unbalanced 1;13 translocation	1	0.91	13	11.82
1q21 deletion syndrome	3	2.73	16	14.55
1q21 deletion syndrome & Trisomy X syndrome	1	0.91	17	15.45
1q21.1 duplication syndrome	1	0.91	18	16.36
1q43-q44 deletion syndrome (1q42.2-q44 deletion/distal monosomy 1q)	1	0.91	19	17.27
22q11.2 deletion syndrome	21	19.09	40	36.36
22q11.2 deletion syndrome & 21q22.13q22.3 duplication	1	0.91	41	37.27
22q11.2 deletion syndrome & first-degree parental consanguiniity	1	0.91	42	38.18
4q26-q35.3 Duplication	1	0.91	43	39.09
5p13 duplication syndrome	1	0.91	44	40.00
5q22.2-q31.2 (24 Mb) deletion (contains 190 genes)	1	0.91	45	40.91

**5q31.3-q35 duplication**	1	0.91	46	41.82
**6p25.3-p25.1 deletion & 9q34.3 duplication due to unbalanced translocation (from a parental balanced translocation)**	1	0.91	47	42.73
**7q11.23 duplication syndrome**	1	0.91	48	43.64
**8p23.1 duplication (involves SOX7 but not GATA4) & Xp22.33/Yp11.32 (SHOX) & 19p13.3 deletion (STK11 and numerous other genes) due to unbalanced translocation (from parental balanced translocation)**	1	0.91	49	44.55
**8p23.1 duplication syndrome**	1	0.91	50	45.45
**Alagille syndrome (20p12 deletion)**	1	0.91	51	46.36
**Emanuel syndrome**	1	0.91	52	47.27
**FOXF1-related disorder/congenital alveolar capillary dysplasia**	1	0.91	53	48.18
**Jacobsen syndrome**	1	0.91	54	49.09
**Kleefstra syndrome (9q34 microdeletion syndrome)**	1	0.91	55	50.00
**Mowat-Wilson syndrome**	2	1.82	57	51.82
**Partial trisomy 5p and 13q11-q12.11 deletion (resulting from unbalanced 5;13 translocation)**	1	0.91	58	52.73
**Recombinant chromosome 8 syndrome**	2	1.82	60	54.55
**Ring chromosome 12 syndrome**	1	0.91	61	55.45
**Supernumerary derivative chromsome 21 & partial trisomy 1q: 47,XX,+der(21)t(1;21)(q31.3;q21.3) causing partial trisomy 21 (pter-q21.3 and partial trisomy 1 (q31.3-qter)**	1	0.91	62	56.36
**Terminal 3pterp22.2 duplication and 12q24.33qter deletion resulting from parental balanced translocation**	1	0.91	63	57.27
**Tetrasomy X**	1	0.91	64	58.18
**Trisomy 13**	2	1.82	66	60.00
**Trisomy 13 (mosaic)**	2	1.82	68	61.82
**Trisomy 13 (mosaic, Robertsonian)**	1	0.91	69	62.73
**Trisomy 16 (mosaic) & Possible UPD16**	1	0.91	70	63.64
**Trisomy 18**	11	10.00	81	73.64
**Trisomy 21**	17	15.45	98	89.09
**Trisomy 21 & Klinefelter syndrome**	2	1.82	100	90.91
**Trisomy 21 (partial) & 2p25.3-p23.1 duplication (unbalanced translocation) due to parental balanced translocation**	1	0.91	101	91.82
**Trisomy 22**	1	0.91	102	92.73
**Turner syndrome**	2	1.82	104	94.55

**Unbalanced chromosome translocation resulting in: 47,XX,+der(9)t(5;9)(p13;q21) leading to 78.5 Mb Duplication at 9pter-q21.13 & 29.1 Mb Duplication at 5pter-p13.3**	1	0.91	105	95.45
**Unbalanced translocation: 46,XX,der(18)t(18:20)(q22;p11.2) -- resulting in 13.2 Mb 18q22.1q23 deletion & 18.3 Mb 20p13p11.23 duplication**	1	0.91	106	96.36
**Unbalanced translocation: 46,XY,t(3;11)(p13;q25)**	1	0.91	107	97.27
**Williams syndrome**	2	1.82	109	99.09
**Wolf-Hirschhorn syndrome**	1	0.91	110	100.00

List of Genetic Diagnoses by Extracardiac Anomaly Status and Genetic Test Type

3. Molecular Diagnoses in Infants with Congenital Heart Defects and No Extracardiac Anomalies

Diagnosis	Frequency	Percent	Cumulative Frequency	Cumulative Percent
ABCC9-related disorder/Cantu syndrome	2	10.00	2	10.00
Adams-Oliver syndrome (DOCK6)	1	5.00	3	15.00
BMPR1A-Related Disorder/Juvenile Polyposis syndrome	1	5.00	4	20.00
CHARGE syndrome	1	5.00	5	25.00
Costello syndrome (HRAS)	1	5.00	6	30.00
DiGeorge syndrome (TBX1)	1	5.00	7	35.00
Ehlers-Danlos syndrome (Vascular)	1	5.00	8	40.00
FLNA-related disorder/cardiac valvular dysplasia	1	5.00	9	45.00
FLT4-related disorder	2	10.00	11	55.00
FOXJ1-related disorder/primary ciliary dyskinesia	1	5.00	12	60.00
GATA4-related disorder & CDK13-related disorder	1	5.00	13	65.00
GATA6-related disorder	1	5.00	14	70.00
MYH7-related disorder (Ebstein anomaly with LVNC)	1	5.00	15	75.00
NOTCH1-related disorder	1	5.00	16	80.00
Neurofibromatosis-Noonan syndrome (NF1)	1	5.00	17	85.00
SETD5-related disorder	1	5.00	18	90.00
TBX1-related disorder	1	5.00	19	95.00
TRRAP-related disorder	1	5.00	20	100.00

List of Genetic Diagnoses by Extracardiac Anomaly Status and Genetic Test Type

4. Molecular Diagnoses in Infants with Congenital Heart Defects with Extracardiac Anomalies

Diagnosis	Frequency	Percent	Cumulative Frequency	Cumulative Percent
ABL1-related disorder	1	2.04	1	2.04
ATRX-related disorder/alpha-thalassemia-X-linked-intellectual disability syndrome	1	2.04	2	4.08
Alagille syndrome (JAG1)	3	6.12	5	10.20
CDH2-related disorder	1	2.04	6	12.24
CHARGE syndrome	7	14.29	13	26.53
Coffin-Siris syndrome (ARID1A)	2	4.08	15	30.61
Diamond-Blackfan anemia (RPL11)	1	2.04	16	32.65
Diamond-Blackfan anemia (RPS24)	1	2.04	17	34.69
Diamond-Blackfan anemia (RPS26)	1	2.04	18	36.73
FLT4-related disorder	1	2.04	19	38.78
GATA6-related disorder	1	2.04	20	40.82
GLI2-related disorder	1	2.04	21	42.86
Holt-Oram syndrome (TBX5)	1	2.04	22	44.90
IFT172-related disorder/Jeune syndrome	1	2.04	23	46.94
KBG syndrome	1	2.04	24	48.98
Kabuki syndrome (KMT2D)	2	4.08	26	53.06
MAST1-related disorder/Mega-corpus-callosum syndrome with cerebellar hypoplasia and cortical malformations	1	2.04	27	55.10
Noonan syndrome (PTPN11)	2	4.08	29	59.18
Noonan syndrome (RAF1)	2	4.08	31	63.27
Noonan syndrome (SOS1)	2	4.08	33	67.35
PACS1-related syndrome/Schuurs-Hoeijmakers syndrome	1	2.04	34	69.39
PEX5-related peroxisomal biogenesis disorder (Zellweger)	1	2.04	35	71.43
PUF60-related disorder/Verheij syndrome	1	2.04	36	73.47
Primary ciliary dyskinesia (DNAH11)	2	4.08	38	77.55
Primary ciliary dyskinesia (DNAH9)	1	2.04	39	79.59

**SIN3A-related disorder**	1	2.04	40	81.63
**SNIP1-related disorder**	2	4.08	42	85.71
**SPECC1L-related hypertelorism syndrome/Teebi hypertelorism syndrome**	1	2.04	43	87.76
**Saethre-Chotzen syndrome (TWIST1)**	1	2.04	44	89.80
**Spinal muscular atrophy (SMN1)**	1	2.04	45	91.84
**Stickler/Marshall syndrome (COL11A1)**	1	2.04	46	93.88
**TBX4-related disorder/heritable PAH/coxopodopatellar syndrome**	1	2.04	47	95.92
**TUBA1A-related disorder**	1	2.04	48	97.96
**TUBB-related tubulinopathy cortical dysplasia with other brain malformations type 6**	1	2.04	49	100.00
