# Supplementary material for: Clinical Decision Analysis of Genetic Evaluation and Testing in 1013 Intensive Care Unit Infants with Congenital Heart Defects Supports Universal Genetic Testing
Source: Genes (Basel). 2024 Apr 18;15(4):505. doi: 10.3390/genes15040505 (PMC11050575; doi:10.3390/genes15040505)

Supplemental Results S2. Proportion of Cytogenetic and Molecular Genetic Disorders across ECA status.

Table SR2-1. Proportion of Cytogenetic and Molecular Genetic Disorders across ECA status.

Extracardiac Anomaly Status	Cytogenetic Abnormality (Column %)	Molecular Abnormality (%)	Cytogenetic & Molecular (>1 Dx)(%)	Clinical Dx (%)	Total (%)
ECA No	57 (33.9)	20 (28.6)	1 (20.0)	0 (0)	78 (31.8)
ECA Yes	111 (66.1)	50 (71.4)	4 (80.0)	2 (100)	167 (68.2)
Total	168	70	5	2	245 (100)

Note: 78/245 patients (31.8%) were ECA-negative and had either a cytogenetic diagnosis or molecular genetic diagnosis. There were no differences in ECA status among patients with all types of genetic diagnoses (Exact p=0.6673).

Figure SR2-1. Proportion of Genetic Diagnoses by Extracardiac Anomaly Status. Low-count genetic diagnosis types were excluded (i.e., those with >1 concurrent genetic diagnoses or clinical diagnoses with negative/inconclusive genetic testing).

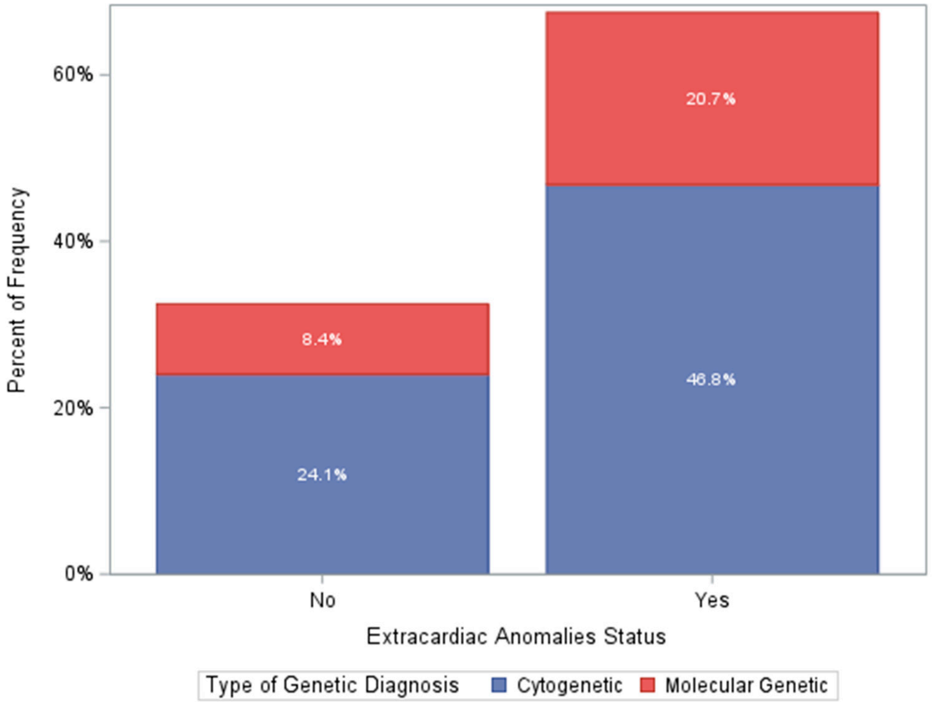

Figure SR2-2. Proportion of Genetic Diagnosis Types. Genetic diagnoses are represented as cytogenetic diagnoses (blue) or molecular diagnoses (red).

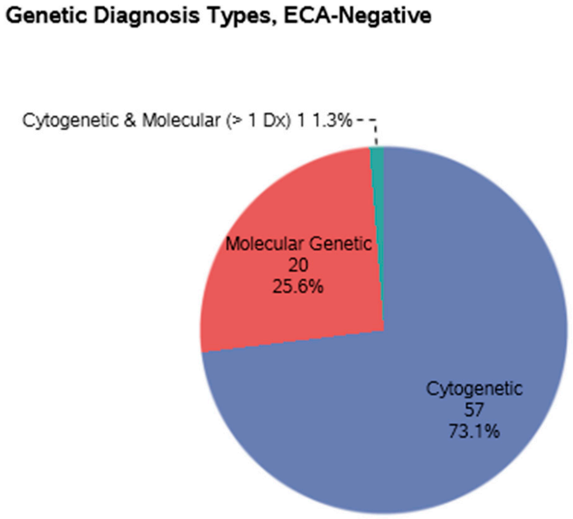

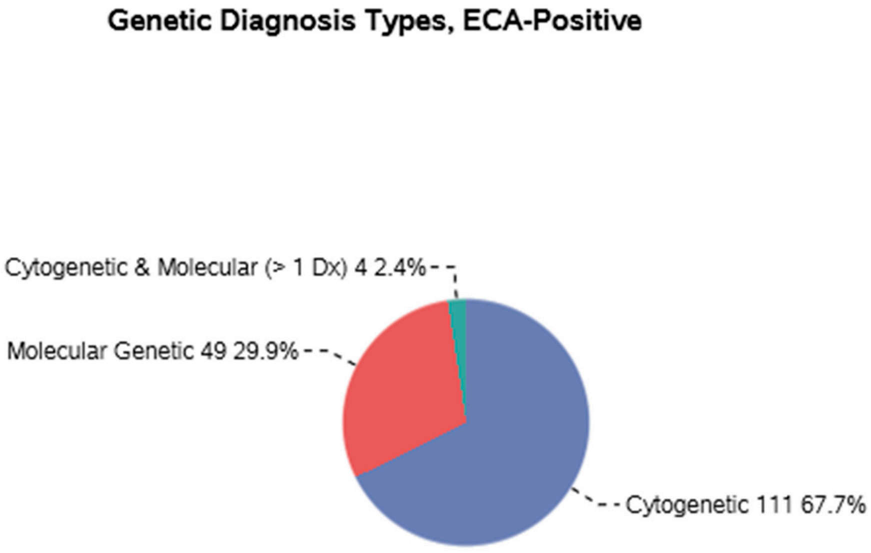

Figure SR2-3. Cohort Results for Genetic Testing. Proportion of genetic testing outcomes are represented separately for cases with or without extracardiac anomalies.

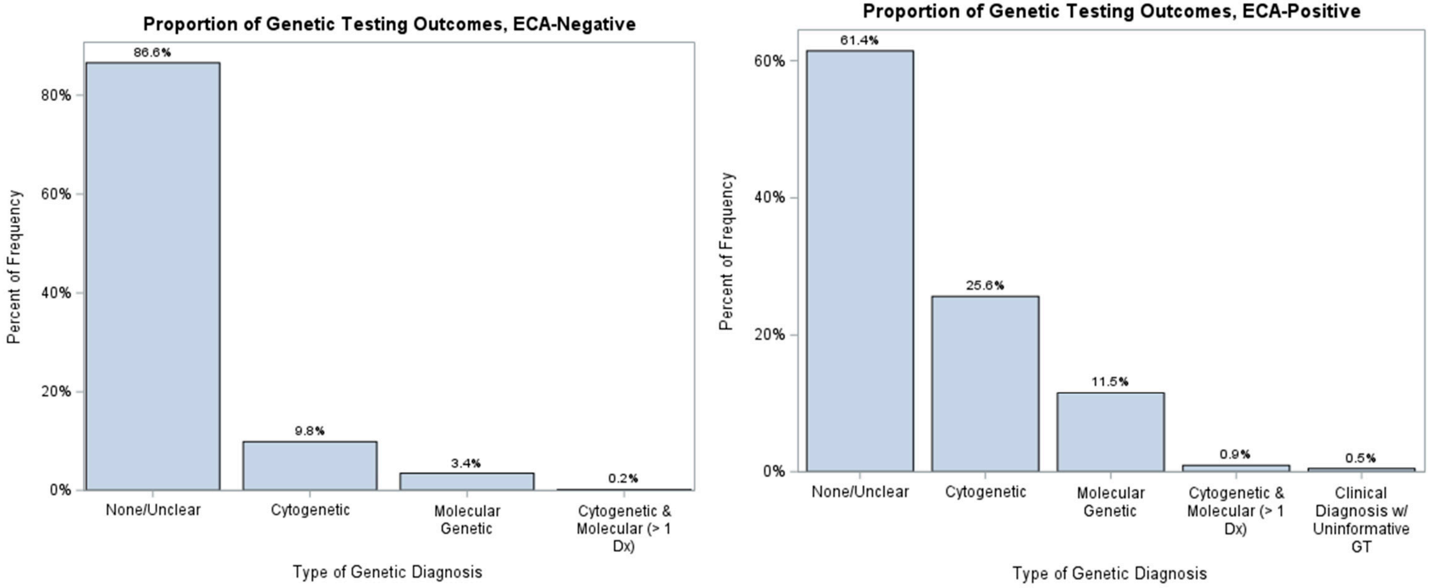

Figure SR2-5. Diagnostic Yield by Genetic Testing Type Stratified by Extracardiac Anomaly Status and Congenital Heart Defect Subtypes: Conotruncal, LVOTO, Complex,

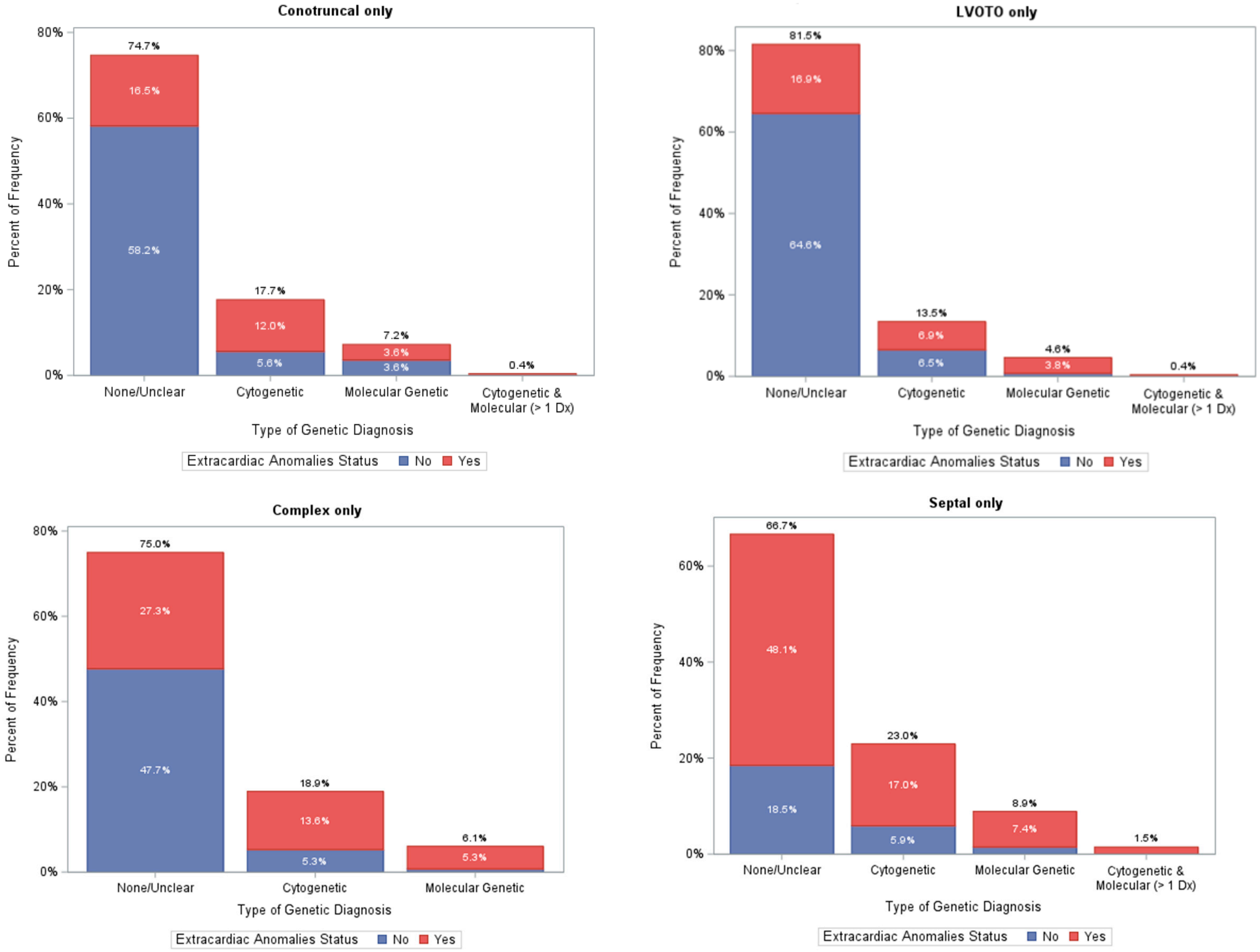

Supplement: Supplementary file 1 [file genes-15-00505-s001.zip › Supplemental-Results_S2.pdf]
