# Supplementary material for: Clinical Decision Analysis of Genetic Evaluation and Testing in 1013 Intensive Care Unit Infants with Congenital Heart Defects Supports Universal Genetic Testing
Source: Genes (Basel). 2024 Apr 18;15(4):505. doi: 10.3390/genes15040505 (PMC11050575; doi:10.3390/genes15040505)
Supplement: Supplementary file 1 [file genes-15-00505-s001.zip › Supplemental-Results_S3.pdf]

Supplemental Results S3. Assessing differences in genetic diagnosis prevalence over the three time periods of our program.

Table SR3-1. Assessing prevalence of genetic diagnosis prevalence over time periods of the clinical program. When restricting the trend analysis to each ECA status respectively, only ECA-negative patients had incremental increases in genetic diagnoses identified over time (p=0.0015). These gains were not seen in the ECA-positive patients. Overall, the composite X^2 test adjusting for ECA status showed that the there was a potential difference across time, though it was marginally significant (p=0.0494).

**Time Period**	**ECA-Negative**		**ECA-positive**	
	Genetic Diagnosis No	Genetic Diagnosis Yes	Genetic Diagnosis No	Genetic Diagnosis Yes
2014-2018	180/195 (92.3%)	15/195 (7.7%)	76/118 (64.4%)	42/118 (35.6%)
2019-2022	265/314 (84.4%)	49/314 (15.6%)	142/235 (60.4%)	93/235 (39.6%)
2023	57/71 (80.3%)	14/71 (19.7%)	49/80 (61.3%)	31/80 (38.8%)
Cochran-Armitage Trend Test one-sided p-value	**p=0.0015**		p=0.2954	
Cochran-Mantel-Haenszel X^2 test, controlling for ECA status	**p=0.0494**			

Table SR3-2. Assessing prevalence of cytogenetic vs. molecular genetic diagnosis types over time periods of the clinical program. When restricting the trend analysis to each ECA status respectively, only ECA-negative patients had incremental increases in genetic diagnoses identified over time (p=0.0356). These gains were not seen in the ECA-positive patients. Overall, the composite X^2 test adjusting for ECA status showed that the there was a difference across time (p=0.0087).

**Time Period**	**ECA-Negative**		**ECA-positive**	
	Cytogenetic Diagnosis	Molecular Genetic Diagnosis	Cytogenetic Diagnosis	Molecular Genetic Diagnosis
2014-2018	14/15 (93.3%)	1/15 (6.7%)	33/39 (84.6%)	6/39 (15.4%)
2019-2022	34/48 (70.8%)	14/48 (29.2%)	57/92 (62.0%)	35/92 (38.0%)
2023	9/14 (64.3%)	5/14 (35.7%)	21/30 (70.0%)	9/30 (30.0%)
Cochran-Armitage Trend Test one-sided p-value	**p=0.0356**		p=0.0651	
Cochran-Mantel-Haenszel X^2 test, controlling for ECA status	**p=0.0087**			
